# Supplementary material for: Cohort profile: Worldwide Collaboration on OsteoArthritis prediCtion for the Hip (World COACH) – an international consortium of prospective cohort studies with individual participant data on hip osteoarthritis
Source: BMJ Open. 2024 Apr 18;14(4):e077907. doi: 10.1136/bmjopen-2023-077907 (PMC11029301; doi:10.1136/bmjopen-2023-077907)
Supplement: Supplementary data [file bmjopen-2023-077907supp001.pdf]

Supplement to:

Cohort profile: Worldwide Collaboration on OsteoArthritis prediCtion for the Hip (World COACH); an international consortium of prospective cohort studies with individual participant data on hip osteoarthritis.

Supplemental Table S1: Indication of available radiographic hip osteoarthritis scores in the included studies at different time points

	**N hips with radiographic hip osteoarthritis score currently available for each follow-up time point**										
**Cohort**	**Baseline**	**2 years**	**4 years**	**5 years**	**6 years**	**7 years**	**8 years**	**10 years**	**12 years**	**15 years**	**20+ years**
CHECK	1,900	1,848		1,746			1,689	1,650			
Chingford	1,268					1,265					x
FORCe	465			x							
JoCoOA	7,750			4,394				2,607		1,355	491
MOST	3,634			3,914							
OAI	8,728		6,712				x				
RS-I	11,239		x				6,929		4,911		2,865
RS-II	4,440		3,314						3,028	x	
RS-III	5,920				4,320						
SOF	11,672						11,672				
TASOAC	1,962							x			
**World COACH**	**58,978**	**1,848**	**10,026**	**10,054**	**4,320**	**1,265**	**20,290**	**4,257**	**7,939**	**1,355**	**3,356**

CHECK = Cohort Hip and Cohort Knee; Chingford = Chingford 1000 women study; FORCe = Femoroacetabular Impingement and Hip Osteoarthritis Cohort Study; JoCoOA = Johnston County Osteoarthritis Project; MOST = Multicenter Osteoarthritis Study; OAI = Osteoarthritis Initiative; RS = The Rotterdam Study; SOF = Study of Osteoporotic Fractures; TASOAC = Tasmanian Older Adult Cohort; World COACH = Worldwide Collaboration on OsteoArthritis prediCtion for the Hip; x = Radiographs available, but not yet scored or score not available yet

Supplemental Table S2: Indication of radiographic hip osteoarthritis development in the included studies

Cohort	Total hips, N	Hips with baseline score and at least 1 follow-up, N (%)	Hips without RHOA at baseline, N (%)	Hips with RHOA at baseline, N (%)	Hips without incident RHOA at follow-up, N (%)	Hips with incident RHOA at follow-up, N (%)
CHECK	2,004	1,827 (91%)	1,627 (89%)	200 (11%)	960 (59%)	667 (41%)
Chingford	2,006	1,265 (63%)	1,108 (88%)	157 (12%)	994 (90%)	114 (10%)
FORCe	465	*NA*	465 (100%)	0 (0%)	*NA*	*NA*
JoCoOA	8,020	4,385 (55%)	3,538 (81%)	847 (19%)	2,792 (79%)	746 (21%)
MOST	6,052	3,885 (64%)	3,486 (90%)	399 (10%)	3,334 (96%)	152 (4%)
OAI	9,592	6,669 (70%)	6,335 (95%)	334 (5%)	6,240 (99%)	95 (1%)
RS-I	16,240	7,192 (44%)	6,783 (94%)	409 (6%)	6,189 (91%)	594 (9%)
RS-II	6,024	3,316 (55%)	3,193 (96%)	123 (4%)	2,943 (92%)	250 (8%)
RS-III	7,878	3,638 (46%)	3,587 (99%)	51 (1%)	3,435 (96%)	152 (4%)
SOF	19,414	11,672 (60%)	11,283 (97%)	389 (3%)	10,846 (96%)	437 (4%)
TASOAC	1,962	*NA*	1,214 (62%)	748 (38%)	*NA*	*NA*
**World COACH**	**79,657**	**43,849 (57%)***	**42,619 (92%)**	**3,657 (8%)**	**37,733 (92%)***	**3,207 (8%)***

*FORCe and TASOAC are not included in this World COACH total for this column, so subtotals may not add up
CHECK = Cohort Hip and Cohort Knee; Chingford = Chingford 1000 women study; FORCe = Femoroacetabular Impingement and Hip Osteoarthritis Cohort Study; JoCoOA = Johnston County Osteoarthritis Project; MOST = Multicenter Osteoarthritis Study; OAI = Osteoarthritis Initiative; RS = The Rotterdam Study; SOF = Study of Osteoporotic Fractures; TASOAC = Tasmanian Older Adult Cohort; World COACH = Worldwide Collaboration on OsteoArthritis prediCtion for the Hip; RHOA = radiographic hip osteoarthritis; NA = Not applicable (no follow-up RHOA scores yet).
