# Supplementary material for: Cohort profile: Worldwide Collaboration on OsteoArthritis prediCtion for the Hip (World COACH) – an international consortium of prospective cohort studies with individual participant data on hip osteoarthritis
Source: BMJ Open. 2024 Apr 18;14(4):e077907. doi: 10.1136/bmjopen-2023-077907 (PMC11029301; doi:10.1136/bmjopen-2023-077907)
Supplement: Author's manuscript [file bmjopen-2023-077907.draft_revisions.pdf]

BMJ Open is committed to open peer review. As part of this commitment we make the peer review history of every article we publish publicly available.

When an article is published we post the peer reviewers' comments and the authors' responses online. We also post the versions of the paper that were used during peer review. These are the versions that the peer review comments apply to.

The versions of the paper that follow are the versions that were submitted during the peer review process. They are not the versions of record or the final published versions. They should not be cited or distributed as the published version of this manuscript.

BMJ Open is an open access journal and the full, final, typeset and author-corrected version of record of the manuscript is available on our site with no access controls, subscription charges or pay-per-view fees (http://bmjopen.bmj.com).

If you have any questions on BMJ Open's open peer review process please email info.bmjopen@bmj.com

BMJ Open

Cohort profile: Worldwide Collaboration on OsteoArthritis prediCtion for the Hip (World COACH); an international consortium of prospective cohort studies with individual participant data on hip osteoarthritis.

Journal:	*BMJ Open*
Manuscript ID	bmjopen-2023-077907
Article Type:	Cohort profile
Date Submitted by the Author:	18-Jul-2023

Complete List of Authors:	van Buuren, Michiel; Erasmus Medical Center, Department of Orthopaedics and Sports Medicine<br>Riedstra, Noortje; Erasmus Medical Center, Department of Orthopaedics and Sports Medicine<br>van den Berg, Myrthe; Erasmus Medical Center, Department of Orthopaedics and Sports Medicine<br>Boel, Fleur; Erasmus Medical Center, Department of Orthopaedics and Sports Medicine<br>Ahedi, Harbeer; University of Tasmania Menzies Institute for Medical Research<br>Arbabi, Vahid; UMC Utrecht, Department of Orthopedics; University of Birjand, Orthopaedic-Biomechanics Research Group, Department of Mechanical Engineering, Faculty of Engineering<br>Arden, Nigel; University of Oxford Nuffield Department of Orthopaedics Rheumatology and Musculoskeletal Sciences<br>Bierma-Zeinstra, Sita; Erasmus Medical Center, Department of General Practice<br>Boer, Cindy; Erasmus Medical Center, Department of Internal Medicine<br>Cicuttini, Flavia; Monash University, Department of Epidemiology and Preventative Medicine<br>Cootes, Timothy; The University of Manchester, Centre for Imaging Sciences<br>Crossley, Kay; La Trobe University School of Allied Health Human Services and Sport, La Trobe Sport and Exercise Medicine Research Centre<br>Felson, David; Boston University School of Medicine,<br>Gielis, Willem Paul; UMC Utrecht, Department of Orthopedics; UMC Utrecht, Department of Radiology<br>Heerey, Joshua; La Trobe University School of Allied Health Human Services and Sport, La Trobe Sport and Exercise Medicine Research Centre<br>Jones, Graeme; University of Tasmania Menzies Institute for Medical Research<br>Kluzek, Stefan ; University of Oxford Nuffield Department of Orthopaedics Rheumatology and Musculoskeletal Sciences<br>Lane, Nancy; University of California Davis School of Medicine, Department of Medicine<br>Lindner, Claudia; The University of Manchester, Centre for Imaging

Sciences
Lynch, John A.; University of California San Francisco, Department of Epidemiology and Biostatistics
Van Meurs, J; Erasmus Medical Center, Department of Orthopaedics and Sports Medicine; Erasmus Medical Center, Department of Internal Medicine
Mosler, Andrea; La Trobe University School of Allied Health Human Services and Sport, La Trobe Sport and Exercise Medicine Research Centre
Nelson, Amanda ; The University of North Carolina at Chapel Hill, Thurston Arthritis Research Center
Nevitt, M; University of California San Francisco, Department of Epidemiology and Biostatistics
Oei, Edwin; Erasmus Medical Center, Department of Radiology & Nuclear Medicine
Runhaar, Jos; Erasmus Medical Center, Department of General Practice
Tang, Jinchi; Erasmus Medical Center, Department of Orthopaedics and Sports Medicine
Weinans, Harrie; UMC Utrecht, Department of Orthopedics; TU Delft, Department of Biomechanical Engineering
Agricola, Rintje; Erasmus Medical Center, Department of Orthopaedics and Sports Medicine

Keywords: Hip < ORTHOPAEDIC & TRAUMA SURGERY, RHEUMATOLOGY, RADIOLOGY & IMAGING, EPIDEMIOLOGY, GENETICS, Risk Factors

SCHOLARONE™ Manuscripts

I, the Submitting Author has the right to grant and does grant on behalf of all authors of the Work (as defined in the below author licence), an exclusive licence and/or a non-exclusive licence for contributions from authors who are: i) UK Crown employees; ii) where BMJ has agreed a CC-BY licence shall apply, and/or iii) in accordance with the terms applicable for US Federal Government officers or employees acting as part of their official duties; on a worldwide, perpetual, irrevocable, royalty-free basis to BMJ Publishing Group Ltd ("BMJ") its licensees and where the relevant Journal is co-owned by BMJ to the co-owners of the Journal, to publish the Work in this journal and any other BMJ products and to exploit all rights, as set out in our licence.

The Submitting Author accepts and understands that any supply made under these terms is made by BMJ to the Submitting Author unless you are acting as an employee on behalf of your employer or a postgraduate student of an affiliated institution which is paying any applicable article publishing charge ("APC") for Open Access articles. Where the Submitting Author wishes to make the Work available on an Open Access basis (and intends to pay the relevant APC), the terms of reuse of such Open Access shall be governed by a Creative Commons licence – details of these licences and which Creative Commons licence will apply to this Work are set out in our licence referred to above.

Other than as permitted in any relevant BMJ Author's Self Archiving Policies, I confirm this Work has not been accepted for publication elsewhere, is not being considered for publication elsewhere and does not duplicate material already published. I confirm all authors consent to publication of this Work and authorise the granting of this licence.

For peer review only

Cohort profile: Worldwide Collaboration on OsteoArthritis prediCtion for the Hip (World COACH); an international consortium of prospective cohort studies with individual participant data on hip osteoarthritis.

M.M.A. van Buuren[1], N.S. Riedstra[1], M.A. van den Berg[1], F. Boel[1], H. Ahedi[2,3], V. Arbabi[4,5], N.K. Arden[6], S.M.A. Bierma-Zeinstra[1,7], C.G. Boer[8], F.M. Cicuttini[9], T.F. Cootes[10], K.M. Crossley[11], D.T. Felson[12], W.P. Gielis[4], J.J. Heerey[11], G. Jones[2], S. Kluzek[6,13], N.E. Lane[14], C. Lindner[10], J.A. Lynch[15], J.B.J. van Meurs[1,8], A. Mosler[11], A.E. Nelson[16], M.C. Nevitt[15], E.H.G. Oei[17], J. Runhaar[7], J. Tang[1], H. Weinans[4,18*], R. Agricola[1].

***Corresponding author**: H.H.weinans@umcutrecht.nl

Affiliations:

1. Department of Orthopaedics and Sports Medicine, Erasmus MC, University Medical Center Rotterdam, Rotterdam, The Netherlands.
2. Menzies Institute for Medical Research, University of Tasmania, Hobart, Tasmania, Australia.
3. Sydney Orthopaedic Research Institute, St Leonards, NSW, Australia .
4. Department of Orthopedics, University Medical Center Utrecht, Utrecht, The Netherlands.
5. Department of Mechanical Engineering, Faculty of Engineering, Orthopaedic-Biomechanics Research Group, Birjand, Iran.
6. Nuffield Department of Orthopaedics, Rheumatology and Musculoskeletal Sciences, Botnar Research Centre, University of Oxford, Oxford, UK.
7. Department of General Practice, Erasmus MC, University Medical Center Rotterdam, Rotterdam, The Netherlands.

For peer review only

8. Department of Internal Medicine, Erasmus MC, University Medical Center Rotterdam, Rotterdam, The Netherlands.
9. Department of Epidemiology and Preventive Medicine, Monash University, Melbourne, Victoria, Australia.
10. Centre for Imaging Sciences, The University of Manchester, Manchester, UK.
11. La Trobe Sport and Exercise Medicine Research Centre, School of Allied Health, Human Services and Sport, La Trobe University, Melbourne, Victoria, Australia.
12. Section of Rheumatology, Boston University School of Medicine, Boston, MA, USA.
13. NIHR Biomedical Research Centre, University of Nottingham, Nottingham, UK.
14. Department of Medicine, School of Medicine, University of California at Davis, Sacramento, CA, USA.
15. Department of Epidemiology and Biostatistics, University of California, San Francisco, CA, USA.
16. Thurston Arthritis Research Center, University of North Carolina at Chapel Hill, Chapel Hill, NC, USA.
17. Department of Radiology & Nuclear Medicine, Erasmus MC, University Medical Center Rotterdam, The Netherlands.
18. Department of Biomechanical Engineering, Delft University of Technology (TU Delft), Delft, The Netherlands.

For peer review only

Abstract

Purpose: Hip osteoarthritis (OA) is a major cause of pain and disability worldwide. Lack of effective therapies may reflect poor knowledge on its aetiology and risk factors, and result in management of end-stage hip OA with costly joint replacement. The Worldwide Collaboration on OsteoArthritis prediCtion for the Hip (World COACH) consortium was established to pool and harmonise individual participant data from prospective cohort studies. The consortium aims to better understand determinants and risk factors for the development and progression of hip OA, to optimise and automate methods for (imaging) analysis, and to develop a personalised prediction model for hip OA.

Participants: World COACH aimed to include participants of prospective cohort studies with ≥200 participants, that have hip imaging data available from at least 2 time points at least four years apart. All individual participant data, including clinical data, imaging (data), biochemical markers, questionnaires, and genetic data, were collected and pooled into a single, individual-level database.

Findings to date: World COACH currently consists of nine cohorts, with 38,021 participants aged 18 to 80 years at baseline. Overall, 71% of the participants were female and mean baseline age was 65.3 ± 8.6 years. Over 34,000 participants had baseline pelvic radiographs available, and over 22,000 had an additional pelvic radiograph after 8–12 years of follow-up. Even longer radiographic follow-up (15–25 years) is available for over 6,000 of these participants.

Future plans: The World COACH consortium offers unique opportunities for studies on the relationship between determinants/risk factors and the development or progression of hip OA, by using harmonised data on clinical findings, imaging, biomarkers, genetics, and

For peer review only

lifestyle. This provides a unique opportunity to develop a personalised hip OA risk prediction model and to optimise methods for imaging analysis of the hip.

Keywords: Hip osteoarthritis, hip morphology, genetics, prediction, epidemiology, risk factors

Strengths and limitations of this study

- The Worldwide Collaboration on OsteoArthritis prediCtion for the Hip (World COACH) consortium brings patients together with a highly qualified and multidisciplinary team of experts and young investigators in the field of hip osteoarthritis, with backgrounds in orthopaedic surgery, rheumatology, physical therapy, general practice, genetics, epidemiology, biostatistics, technical medicine, biomechanical engineering, radiology, imaging science, and artificial intelligence.
- The World COACH consortium is unique for having harmonised individual participant data on clinical measurements, radiological imaging, biochemical markers, lifestyle and diet, comorbidities, medication, physical and cognitive functioning, quality of life and genetics from over 38,000 people, both from the general population as well as from specific populations at risk for hip osteoarthritis.
- The World COACH consortium has sequential hip radiography available for each participant with a follow-up duration ranging from 5 to over 25 years.
- The main limitations of the consortium are the geographic origins of the included cohorts (Western world) and the heterogeneity in collected data by the cohorts, which may limit the possibilities of harmonisation.

For peer review only

Introduction

Osteoarthritis (OA) is a common disease and a leading cause of disability in adults(1). Over 500 million people are affected by OA worldwide, leading to a global prevalence of around 7%(2). The forecast for OA is alarming; with an ageing population, the prevalence is expected to rise dramatically in the coming decades. The direct healthcare costs of OA in various high-income countries account for 1% - 2.5% of the gross domestic product(3, 4). OA can affect any joint and is most prevalent in the knee and hip, where it also leads to the greatest physical disability(5). Due to a subsequent decrease in physical activity, hip OA also leads to more comorbidities and a higher age-adjusted mortality(6).

Despite the tremendous burden of hip OA, there is no cure available. Therefore, current strategies focus on symptomatic treatment with only a modest effect(7). This may partly result from a lack of knowledge on the aetiology, pathophysiology and risk factors of hip OA. Hip OA is a heterogeneous disease in which the risk factors and aetiology can differ widely from patient to patient. The lack of knowledge regarding person-specific risk factors for hip OA makes efficient and effective preventative and treatment strategies challenging, if not impossible, thus only one-size-fits-all treatment options for hip OA are available to date. Still, some risk factors for hip OA have been identified on a group level, such as age, gender(5), genetics(8), race(9), and hip morphology(10, 11). However, each risk factor has only weak or even conflicting associations with hip OA, they have mainly been studied in single, heterogeneous cohorts, and are typically studied separately from each other. These single studies are underpowered to predict the risk of hip OA on an individual level.

The prospective cohort study design is ideal to better understand which individuals are at risk of developing hip OA and of progressing to end-stage disease. Harmonising data from multiple cohort studies into an individual participant-level database provides a large sample size, which allows for person-specific risk estimates. Further, large sample sizes and diverse cohorts from all over the world improve the generalisability of the findings(12). To meet this need, the Worldwide Collaboration on OsteoArthritis prediCtion for the Hip (World COACH) consortium was established in 2018. The consortium aims to better understand risk factors for the development or progression of hip osteoarthritis, and to optimise and automate methods for analysing radiological images of the hip. This will be pursued through studying multiple research questions within the consortium.

CONSORTIUM DESCRIPTION

Cohort inclusion and consortium establishment

Prospective cohort studies were considered eligible if they had hip radiography – and optionally computed tomography (CT) and/or magnetic resonance imaging (MRI) – available at two or more points in time, at least four years apart, and if they had a minimum of 200 participants at baseline. A systematic literature search was conducted in Embase, Ovid MEDLINE and Cochrane CENTRAL to identify all studies that fulfilled the inclusion criteria. The search was first carried out in 2017 and was repeated in October 2020 and again in March 2023. Titles and abstracts were screened independently by two researchers (MvB and RA), and all described cohorts were further investigated, both by reading the full texts of the screened references and by additional internet searches. A PRISMA flow

eview only

For peer review only - http://bmjopen.bmj.com/site/about/guidelines.xhtml

diagram of the search and inclusion process is presented in **Figure 1**. In summary, we screened a total of 1,970 records by title and abstract, of which we assessed 195 records in detail. We identified 40 study cohorts, 10 of which we considered eligible for the consortium. Investigators from the eligible cohorts were contacted and asked to collaborate. To date, 9 cohorts have been included in the consortium(13-21), and contact has been initiated with the remaining eligible cohort(22). The systematic search will be repeated every 2 years to identify newly eligible cohorts.

After the first search, the initiator of the World COACH consortium (RA) contacted the principal investigators of the 9 identified cohorts to discuss the consortium's aims. A live meeting with principal investigators of 8 cohorts, as well as other individuals interested in participating in the consortium, was held during the OsteoArthritis Research Society International (OARSI) world congress in Liverpool, 2018. During this meeting, the overall aims of the consortium were presented, and an inventory of the support for initiating the consortium was assessed. With unanimous support for the consortium, we decided to establish this initiative. Legal agreements for data sharing were drafted and executed. Since 2018, quarterly meetings have been held with the collaborators to determine the aims and research plans of the consortium.

Description of the cohorts

A summary of the included cohorts can be found in **Table 1**. All included studies are prospective cohort studies with a minimum of 5 and a maximum of more than 25 years of follow-up. Some cohorts still have ongoing data collection(13, 16, 20). The earliest data

For peer review only

collection in any cohort started in 1986(14), and the most recent baseline data collection started in 2016(16). Most included cohorts are population-based studies. Exceptions are the CHECK cohort, in which participants had hip or knee complaints, MOST and OAI, which both included individuals with or at high risk of knee OA, and FORCe, which included participants with hip and/or groin pain. At least 6 cohorts included non-white individuals, and 6 cohorts studied both men and women. The total World COACH population includes participants from 3 continents. Across cohorts, participants were aged 18 to 80 years at enrolment. The aims and methods of each individual cohort are shortly described below, emphasising those characteristics that correspond with the consortium's inclusion criteria (e.g. radiographic protocols are highlighted, but not CT or MRI).

For peer

Cohort Hip and Cohort Knee (CHECK) study

The CHECK study was a multi-centre prospective cohort study in the Netherlands that ran from 2002 until 2015 for a total of 10 years follow-up(21). The aim was to study the course, prognosis and underlying mechanisms of early symptomatic OA. The study included 1,002 participants aged 45–65 years, with a first episode of pain in the hip and/or knee. Participants were eligible if they had not yet visited a general practitioner (GP) or were within 6 months of their first visit to the GP for these symptoms, or if they had never visited a GP before for these symptoms, and if there was no other diagnosis that could explain the symptoms at the time of inclusion. Standardised weight-bearing anteroposterior (AP) hip and pelvic radiographs, using a wedge to get the hips in 15° internal rotation, were obtained at baseline and at 2, 5, 8 and 10 years of follow-up.

The Chingford 1000 women study

The Chingford study is a population-based prospective cohort study that aimed to assess musculoskeletal disease in the female population(15). It ran from 1989 to 2010, having over 20 years of follow-up. The study included 1,003 asymptomatic female participants, aged 45–64 years, who were recruited from the registry of a general practice in Chingford, London, UK. Standardised supine AP pelvic radiographs, using a small sand bag under the knees to minimise hip rotation, were obtained at year 2, 8 and 20 of follow-up(23).

Femoroacetabular impingement and hip OsteoaRthritis Cohort (FORCe) study

The FORCe study is an ongoing prospective cohort study aiming to evaluate changes in hip joint structure in sub-elite soccer and Australian football players with hip and/or groin pain, with a focus on early hip OA features(13). The study included 239 participants, aged 18-50 years, who were recruited through advertisements at sporting venues and from orthopaedic, sports medicine or physical therapy clinics. At baseline, all participants underwent standardised supine AP pelvic radiographs with the feet in 15° internal rotation using a positioning aid, and MRI of the hips. The study is currently inviting participants for a 5-year follow-up visit, that comprises pelvic radiography and MRI according to the same standardised protocols.

Johnston County Osteoarthritis Project (JoCoOA)

The JoCoOA is a population-based cohort study with up to 30 years of follow-up(19, 24). Its aim has been to examine the incidence, prevalence and progression of osteoarthritis in African American and white men and women in a rural county. The study started in 1991

and data collection ended in 2018. Participants, all non-institutionalised black and white men and women, were drawn from the population of Johnston County, North Carolina, USA. The study included 4,337 participants aged 45 years and older. Standardised supine AP pelvic radiographs with the feet in 15° internal rotation were obtained at baseline, and then every 5–6 years.

Multicenter Osteoarthritis Study (MOST)

MOST is a multicentre prospective cohort study in the USA that started in 2003 and has followed participants for 20 years so far (20). The aim was to study risk factors for the development and progression of knee osteoarthritis and knee pain. Two centres in Birmingham (Alabama) and Iowa City (Iowa) recruited participants with pre-existing knee OA or those at high risk for knee OA from the general population. The study included 3,026 individuals aged 50–79 years in its initial phase, with a new cohort of 1,500 individuals included in 2016-2018. A standardised weight-bearing AP full-limb radiograph of the lower extremities (including the pelvis) with the tibial tubercles facing forward and the X-ray beam centred at the knee was obtained at baseline, and again at 5 years of follow-up.

Osteoarthritis Initiative (OAI)

The OAI study was a multicentre prospective cohort study of knee OA in the USA(18, 25). OAI aimed to provide resources to enable a better understanding of prevention and treatment of knee OA. It was initiated in 2002 and the entire cohort finished its 8-year follow-up in 2015, but the follow-up continues for certain subsets of participants. The OAI study has included 4,796 participants with pre-existing knee OA or those at high risk for

developing knee OA, from the general populations of Baltimore (Maryland), Columbus (Ohio), Pittsburgh (Pennsylvania), and Pawtucket (Rhode Island). Participants were aged 45–79 years at enrolment. Standardised weight-bearing AP pelvic radiographs using a v-shaped foot-positioning frame to get the feet in 5° of internal rotation were obtained at baseline, 4 years, and 8 years follow-up.

The Rotterdam Study (RS)

The RS is an ongoing prospective population-based cohort study in a district of the city of Rotterdam, the Netherlands(26). It aims to address determinants and occurrence of cardiovascular, neurological, musculoskeletal, ophthalmologic, psychiatric, and endocrine diseases in the elderly. After the pilot in 1989, the study started recruiting in 1990, and it currently has over 25 years of follow-up. Up to 2008, the study had included 14,926 participants aged 45 years and over, divided into three sub-cohorts from different enrolment periods, namely RS-I, RS-II and RS-III. Recruitment of a fourth sub-cohort (RS-IV) started in 2016 and has recently been finished. Data from RS-IV will also be included once they fulfil the inclusion criteria. Standardised weight-bearing AP pelvic radiographs with the feet in 10° internal rotation were obtained at baseline, and then approximately every 5 years.

Study of Osteoporotic Fractures (SOF)

SOF is a multi-centre prospective population-based cohort study of community-dwelling women aged ≥65 years(14). The primary purpose of SOF was to describe risk factors for osteoporotic fractures. Women were recruited from 4 metropolitan areas in the USA:

Baltimore (Maryland), Pittsburgh (Pennsylvania), Minneapolis (Minnesota) and Portland (Oregon). The original cohort started in 1986 and included 9,704 mostly Caucasian women who had not undergone bilateral hip replacement and were able to walk without assistance. The cohort has over 20 years of prospective data about osteoporosis. Standardised supine AP pelvic radiographs with the hips in 15–30° internal rotation were obtained at baseline and after 8 years of follow-up.

Tasmanian Older Adults Cohort (TASOAC) study

The TASOAC study is an ongoing prospective population-based cohort study of 1,099 community-dwelling men and women, aged 50–80 years, who were randomly selected from the electoral roll in Southern Tasmania(17). The study aimed to identify factors associated with the development and progression of OA in multiple joints, including the hip. Participants were excluded if they were institutionalised or if they reported contraindication for MRI. Enrolment started in 2002 and the cohort had follow-up moments at approximately 2.7 years, 5 years and 10 years. Standardised weight-bearing AP pelvic radiographs with the feet in 10° internal rotation were obtained at baseline and after 10 years of follow-up. A subgroup (n=250) had MRI of the right hip in the sagittal plane at 2.7 and 5 years follow-up(27).

DATA HARMONISATION

Retrospective harmonisation is an intricate process, considering few original studies have used identical collection methods and procedures. Our harmonisation process was based both on expert opinion within the consortium, as well as on the Maelstrom Research

guidelines for rigorous retrospective data harmonisation(28). The first steps of this approach regarding defining research questions and objectives, and selecting studies have already been described above.

Defining the DataSchema

We started by analysing the present literature on the included studies (e.g. study protocols, published papers) to evaluate sources of study heterogeneity. The next step was to define variables and evaluate the harmonisation potential. All available variables from individual studies within the consortium were identified and systematically entered in a DataSchema(28), categorised in thirteen sections: demographic data, physical examinations and anthropometry, radiographic measurements of OA, questionnaires, family history, procedures, biospecimens, lifestyle and diet, comorbidities, medication, physical and cognitive functioning, quality of life, and genetics. This allowed us to evaluate comparability between studies. Next, all data was catalogued based on their characteristics. All similar variables that indicate the same measurement were grouped together and renamed using a common pooled variable. Finally, the process of data harmonisation was initiated, for which we used and will continue to use one of the established approaches, depending on the data(28):

- **Simple calibration model**: will be used to transform continuous variables into new continuous variables (e.g. transforming height in inches to height in centimetres). The distribution of the values will be compared across cohorts to assess for differences within the measurement.

- **Algorithmic transformation**: will be used to harmonise continuous or categorical variables with combinable ranges or categories (e.g. race or ethnicity, education level).
- **Standardisation model**: will be used to harmonise the same constructs measured with different scales, when there are no bridging items available (e.g. two independent questionnaires on hip symptoms)
- **Latent variable model**: will be used to harmonise variables with different scales that have some bridging items (e.g. OA grade based on the Kellgren-Lawrence (KL), Croft or OARSI atlas classification, which all contain items such as joint space narrowing and osteophytes).

Data storage and processing

After establishing data transfer agreements with each included cohort, all required individual participant data were transferred to a central server. Variables were then prepared to be entered into a relational database using the Observational Medical Outcomes Partnership (OMOP) Common Data Model (CDM) structure(29, 30). The CDM is a 'person-centric' model and is optimised for observational research purposes such as identifying patient populations with certain outcomes (such as hip OA), characterisation of these populations for various parameters (including risk factors), and predicting the occurrence of the outcome in individuals. Variables were clustered in domains using the CDM's standardised vocabularies. Although the variables were mapped to the standardised vocabularies, we also stored the original source values, to ensure that all data entries can be traced when locating or preventing unforeseen errors. The OMOP CDM does not require

specific software and can be realised in any relational database software. We currently use an advanced open-source relational database system (PostgreSQL version 15.2, PostgreSQL Global Development Group) which uses the SQL data definition and query language. The stored variables from each cohort and their individual participants include demographics and follow-up visits, along with measurements and procedures performed at each visit. This original data collection setup is used within the relational database model, which contains seven linked tables (**Figure 2**). The person table contains demographics of the included individual, such as biological sex, year of birth, and the originating cohort identification number, which links to the descriptive cohort table. The visit occurrence table contains the different time points and study sites (where available) at which data was collected from each individual. The measurement and procedure occurrence tables contain harmonised and newly generated World COACH variables for each specific follow-up visit (e.g. harmonised radiographic hip OA score based on available KL or modified Croft grades). The harmonisation steps are documented and harmonised values are linked to their source value through the harmonisation key table.

Outcome measures

The main outcome is the development of hip OA within the various follow-up periods, although there are several outcomes of interest for secondary analyses. Hip OA will be defined structurally by radiological indices, clinically by pain and/or functional indices, and by a combination of these two. Radiographs are the only validated and recommended imaging modality to investigate hip OA as a structural outcome(31, 32). Pelvic radiographs have been read for the presence and severity of radiographic OA using either the KL

classification(33, 34), the (modified) Croft classification(35-37), or the atlas of individual radiographic features in osteoarthritis (OARSI atlas)(38). The inclusion criterion of having hip radiography available at two or more points in time, at least four years apart, was set to determine the presence or absence of radiographic hip OA at both time points. This is necessary to distinguish between incident radiographic hip OA (in case of no radiographic hip OA at baseline) or progression of radiographic hip OA (in case of radiographic hip OA at baseline). Different pain scores, such as visual analogue scales (VAS), Western Ontario and McMaster Universities Osteoarthritis Index (WOMAC) pain scale, the Hip Disability and Osteoarthritis Outcome Score (HOOS), and other scores will be harmonised into a single pain index if and where possible.

FINDINGS TO DATE

As of submission, the World COACH database contains data on 38,021 individuals. The mean age of all World COACH participants at baseline was 65.3 ± 8.6 years and the study sample consists of 27,010 (71.0%) women (**Table 2**).

To study the research questions of the World COACH Consortium, the project has five separate work packages. The first work package is Methodology, of which the goal is to discover, optimise, automate and validate new methods in OA research, such as an automated pipeline for hip morphological analyses and developing algorithms for the detection of radiographic hip OA. This includes the application of artificial intelligence (AI). Hip Morphology is the second work package and it focuses on investigating associations between hip morphology (such as acetabular dysplasia, pincer morphology, cam

eview only

For peer review only - http://bmjopen.bmj.com/site/about/guidelines.xhtml

morphology and general hip shape as defined by statistical shape modelling) and hip OA. The third work package is Genetics, of which the aim is to study associations between genetics, hip morphology, environmental factors, and OA, by applying Genome-Wide Associations Studies (GWAS) among other methods. The fourth work package is Clinical Measures, comprising physical examinations, questionnaires, quality of life and blood and urine samples. The aim is to study the associations between these measures and the development of hip OA. Finally, the fifth work package combines data and results from all other work packages to develop a personalised risk prediction model for the development and progression of hip OA, using both conventional and AI-driven methods.

Currently, analyses are being conducted across all work packages. Furthermore, the World COACH research team is making efforts to expand the consortium with other potential cohort studies.

Strengths and limitations

The main strength of the World COACH consortium is its rich variety of harmonised, individual participant data from all available prospective cohort studies on hip OA worldwide. Although this offers significant challenges, it has the potential to improve the generalisability of our findings. The large sample size offers unique opportunities to study the relationship between determinants and risk factors and the development and progression of hip OA on an individual level, as well as the identification of high-risk subgroups. It also allows for analysis of interactions between these factors, such as the effect of obesity across different hip shape variations. This will allow for the development

For peer review only

of the first person-specific risk estimation of developing hip OA. This personalised model can in turn be used to identify both high-risk individuals and the factors that contribute to this risk for each specific person. In turn, this provides opportunities for future studies on prevention and individualised OA treatment. Furthermore, the World COACH consortium strives to offer solutions to some of the greatest epidemiological issues in terms of hip OA research by testing, automating, and validating methodological issues related to image analysis, with the potential of providing a benchmark for imaging analysis in hip OA research. Finally, the extensive dataset allows for investigating an array of secondary research questions along with the main aims of the consortium.

The strength of a relational database is that it is possible to enrich the existing consortium data with data from new cohorts once they meet the inclusion criteria, without the need to restructure the datasets. The flexible structure of relational databases allows for seamless expansion to handle increasing volumes of data and it can easily adapt to frequent updates or deletions.

Limitations of the consortium include the limited geographic selection of cohorts from the Western world (Australia, Europe and the United States). To date, no cohorts have been eligible or included from Africa, Asia or South America, which limits the generalisability of findings. The World COACH consortium is also limited by the heterogeneity in collection of variables by the cohorts, which was inherently done in slightly different ways. This requires harmonisation of variables, which is mainly based on (potentially subjective) expert-based criteria. On the other hand, pooling of the data creates far greater statistical power than previously possible. Finally, although a subset of the data consists of 3-dimensional imaging data such as CT or MRI, most analyses will be performed using plain

For peer review only

AP pelvic radiographs. Still, radiographs probably contain valuable (hidden) predictive information for hip OA, and they are extensively used in daily clinical practice. Radiographs are also the only valid method to diagnose structural hip OA so far and are a simple and inexpensive tool for use in large clinical studies.

Collaboration

We will provide a harmonised database containing all prospective data on hip OA. We encourage the use of data by third parties, although this is subject to approval by the steering committees of the World COACH consortium and the participating cohorts, as well as to legal boundaries regarding data ownership. To streamline the processing of third-party requests, we have developed a standardised data request form that can be distributed and reviewed uniformly. This will ensure consistency in the way data requests are handled within World COACH.

Our approach to data storage involves the Findable, Accessible, Interoperable and Reusable (FAIR) principles(39). This will be achieved by using unique and persistent identifiers, by adhering to the Observational Health Data Sciences and Informatics (OHDSI) terminology where possible, and by implementing standardised access protocols to make data available upon request. By adhering to the FAIR principles, we aim to promote collaboration and transparency to advance scientific research in the field of hip OA and beyond. The relational database supports data storage that is compatible with other data sources and formats, enabling seamless integration.

Finally, the project will be overseen by two committees: a steering committee and an advisory committee, which have quarterly meetings regarding the consortium. Both

For peer review only

committees consist of a diverse team of experienced researchers and clinicians in the areas of osteoarthritis, rheumatology, epidemiology and image processing. Their combined expertise will provide valuable guidance and ensure the project's success.

More information on the consortium and on data requests can be obtained from the website: www.worldcoachconsortium.com.

Patient and public involvement

A patient and public committee (PPC) is being formed to ensure that the wider public is represented in World COACH. World COACH aims to ensure that all projects are relevant, meaningful and have impact on the people and patients it aims to serve. This includes not only hip OA patients, but also families, caregivers and members of the general public. The PPC will be involved in prioritising research questions and in helping to shape the long-term vision of World COACH with particular consideration for the interests of the public and hip OA patients. Our goal is to engage with a relevant population by promoting our project at various events. Additionally, we have made our research team accessible to the public through the World COACH website, where individuals can contact us directly via email, and through public meetings such as a local "OA cafe". We actively encourage such interactions in our presentations to foster engagement and promote greater understanding of our research to the public, and for the team to better understand what is relevant and important to patients.

Acknowledgments

For peer review only

We would like to thank all participants of the cohort studies that are involved in the World COACH consortium. We gratefully acknowledge all international organisations that collaborated with the cohort studies in World COACH, as well as the Osteoarthritis Research Society International (OARSI) for endorsing the World COACH consortium. We thank the (non-profit) funding bodies who financially support the World COACH consortium: the Dutch Arthritis Society (grant no. 18-2-203 and 21-1-205), the Dutch Research Council (NWO Veni grant scheme no. 09150161910071) and the Erasmus MC, University Medical Center, Rotterdam (Erasmus MC Fellowship). For the purposes of open access, the authors have applied a CC BY public copyright licence to any Author Accepted Manuscript version arising from this submission.

CHECK: The CHECK study was initiated by the Dutch Arthritis Society and performed within: Erasmus Medical Center Rotterdam; Kennemer Gasthuis Haarlem; Leiden University Medical Center; Maastricht University Medical Center; Martini Hospital Groningen/Allied Health Care Center for Rheum. and Rehabilitation Groningen; Medical Spectrum Twente Enschede/Ziekenhuisgroep Twente Almelo; Reade, formerly Jan van Breemen Institute/VU Medical Center Amsterdam; St.Maartens-kliniek Nijmegen; University Medical Center Utrecht and Wilhelmina Hospital Assen.

Chingford: We would like to thank all the participants of the Chingford Women Study, Professor Nigel Arden, Professor Tim Spector, Dr Deborah Hart, Mr Gem Lawson, Maxine Daniels and Alison Turner for their time and dedication and Arthritis Research UK for their funding support to the study and the Oxford NIHR Musculoskeletal Biomedical Research Unit for funding contributions.

For peer review only

FORCe: We would like to thank the staff at Imaging at Olympic Park and Qscan radiology clinics who continue to assist in study data collection, and the study participants. The FORCe study was funded by two National Health and Medical Research Council (NHMRC) of Australia project grants (GNT1088683 and GNT1156674).

JoCoOA: Support for data from the Johnston County Osteoarthritis Project was provided in part by: the Center for Disease Control and Prevention (CDC) U01DP006266 and U01DP003206; Association of Schools of Public Health/ CDC S043, S1734, S3486; and National Institutes of Health/National Institute of Arthritis and Musculoskeletal and Skin Diseases P60AR30701, P60AR049465, P60AR064166, and P30AR072580.

MOST: The MOST study was funded by the National Institutes of Health – National Institute on Aging grants AG19069 (Michael Nevitt, University of California, San Francisco) AG18820 (David Felson, Boston University) AG18947 (Cora Lewis, University of Alabama at Birmingham) and AG18832 (James Torner, University of Iowa).

OAI: The OAI is a public-private partnership comprised of five contracts (N01-AR-2-2258; N01-AR-2-2259; N01-AR-2-2260; N01-AR-2-2261; N01-AR-2-2262) funded by the National Institutes of Health, a branch of the Department of Health and Human Services, and conducted by the OAI Study Investigators. Private funding partners include Merck Research Laboratories; Novartis Pharmaceuticals Corporation, GlaxoSmithKline; and Pfizer, Inc.

Rotterdam Study: The Rotterdam Study is funded by Erasmus University Medical Center and Erasmus University, Rotterdam, The Netherlands Organisation for Health Research and Development (ZonMw), the Research Institute for Diseases in the Elderly (RIDE), the Ministry of Education, Culture and Science, the Ministry for Health, Welfare and Sports, the European Commission (DG XII), and the Municipality of Rotterdam. The authors are

For peer review only

For peer review only - http://bmjopen.bmj.com/site/about/guidelines.xhtml

grateful to the study participants, the staff from the Rotterdam Study and the participating general practitioners and pharmacists.

SOF: The Study of Osteoporotic Fractures (SOF) is supported by National Institutes of Health funding. The National Institute on Aging (NIA) provides support under the following grant numbers: R01 AG005407, R01 AR35582, R01 AR35583, R01 AR35584, R01 AG005394, R01 AG027574, and R01 AG027576

TASOAC: The TASOAC study was supported by the National Health and Medical Research Council of Australia, Tasmanian Community Fund, Masonic Centenary Medical Research Foundation, Royal Hobart Hospital Research Foundation and Arthritis Foundation of Australia.

Funding:

The World COACH consortium itself has been funded through research grants by the Dutch Arthritis Society (grant no. 18-2-203 and 21-1-205), the Dutch Research Council (NWO Veni grant scheme no. 09150161910071), and Erasmus MC, University Medical Center Rotterdam (Erasmus MC Fellowship).

MvB is funded by the Dutch Arthritis Society (research grants 18-2-203 and 21-1-205) and by an Erasmus MC Fellowship grant. NR, MvdB and FB are funded by the Dutch Arthritis Society (research grant 21-1-205). NA is funded by the Versus Arthritis Centre for Sport, Exercise & Osteoarthritis Research. SBZ is funded through independent research grants by the European Research Council (ERC), the Dutch Arthritis Society, and The Netherlands Organisation for Health Research and Development (ZonMw). CB and JvM are funded by the Dutch Arthritis Society (LLP-34). TC is funded by research grants from the Engineering

For peer review only

and Physical Sciences Research Council (EPSRC) UK, the Medical Research Council (MRC) UK, and the Wellcome Trust. KC and JH are funded by the National Health and Medical Research Council (NHMRC) Australia (GNT GNT1088683), AM is funded by an NHMRC Australia Early Career Fellowship (GNT1156674). DF is funded by a research grant from the National Institutes of Health (NIH) [AR072571]. CL is funded by a research grant from the MRC UK (MR/S00405X/1) as well as a Sir Henry Dale Fellowship jointly funded by the Wellcome Trust and the Royal Society (223267/Z/21/Z). This research was funded in whole, or in part, by the Wellcome Trust [Grant number 223267/Z/21/Z]. AN is funded by the Centers for Disease Control and Prevention (CDC) [U01DP006266 and U01DP003206; Association of Schools of Public Health/ CDC S043, S1734, S3486], and the NIH and National Institute of Arthritis and Musculoskeletal and Skin Diseases (NIAMS) [P60AR30701, P60AR049465, P60AR064166, and P30AR072580]. MN is funded by research grants from the NIH. JT is funded by the China Scholarship Council (CSC). HW is funded by research grants from Interreg, Kansen voor West, NWO, the Innovative Medicines Initiative (IMI), and the Dutch government. RA is funded by the Dutch Arthritis Society (research grants 18-2-203 and 21-1-205), the Dutch Research Council (NWO Veni grant scheme no. 09150161910071), and Erasmus MC, University Medical Center Rotterdam (Erasmus MC Fellowship).

For peer review only

Competing interests statement:

GJ reports personal fees from Novartis outside the submitted work. SBZ reports consulting fees from Pfizer Infirst Healthcare and personal fees for being a Deputy Editor for *Osteoarthritis and Cartilage* outside the submitted work. CL and TC report a patent for an

image processing apparatus and method for fitting a deformable shape model to an image using random forest regression voting. CL reports licensing royalties for this patent from Optasia Medical outside the submitted work. AN is an associate editor for *Osteoarthritis and Cartilage* and is on the OARSI Board of Directors outside the submitted work. AM is on the Editorial Board for the *British Journal of Sports Medicine* and the *Journal of Science and Medicine in Sport* outside the submitted work. HW reports being a minority shareholder of Uplanner BV and Replasia BV outside the submitted work.

Contributors:

RA initiated and worked on the conceptual design of the study. MvB and RA identified eligible cohorts and contacted cohort investigators for collaboration. MvB and RA collected the existing cohort data. MvB, NR, MvdB, FB, JT, and RA have worked on the database and on the harmonisation process. All authors critically revised the manuscript and contributed to interpretation of the data. All authors read and approved the final version of the manuscript.

For peer review only

TABLES AND FIGURES

Table 1: Information and characteristics of the included cohorts.

Cohort	Abbreviation	Recruitment	N baseline participants	N baseline pelvic / hip radiographs	Age at enrolment	Races included	Follow-up time	Follow-up status
Cohort Hip and Cohort Knee	CHECK	First visit with hip/knee pain	1,002	1,002	45 – 65 years	W, B, A, O	10 years	Completed
Chingford 1000 women study	Chingford	Women from general population	1,003	1,003	45 – 67 years	NS	19 years	Completed
Femoroacetabular Impingement and Hip Osteoarthritis Cohort Study	FORCe	Sub-elite soccer and Australian football players with hip and/or groin pain	239	239	18 – 50 years	NS	5 years	Ongoing
Johnston County Osteoarthritis Project	JoCoOA	General population	4,337	4,004	≥45 years	W, B	25+ years	Completed
Multicenter Osteoarthritis Study	MOST	Existing knee OA or at high risk	3,026	3,008	50 – 79 years	W, B, A, O, NS	7 years	Ongoing
Osteoarthritis Initiative	OAI	Existing knee OA or at high risk	4,796	4,771	45 – 79 years	W, B, A, O, NS	8 years	Completed
The Rotterdam Study	RS	General population	14,926	11,147	≥ 45 years	W, B, A, Mix	25 years	Ongoing

For peer review only

Study of Osteoporotic Fractures	SOF	Women from general population	10,366	8,291	≥ 65 years	W, B	8 years	Completed
Tasmanian Older Adult Cohort	TASOAC	General population	1,099	1,099	50 – 80 years	W, NS	10 years	Completed
**Summary:**	**World COACH**	**Mixed populations**	**40,794**	**34,257**	**18 – 80 years**	**W, B, A, O, Mix**	**5 – 25 years**	**Ongoing / Completed**

Race abbreviations: W = white, B = black, A = Asian, O = other, NS = not specified, Mix = admixture

For peer review only

Table 2: Baseline characteristics of the included cohorts.

Cohort	N participants with data available	Mean (SD) age, years	Mean (SD) height, cm	Mean (SD) weight, kg	Mean (SD) BMI, kg/m^2	% Female
CHECK	1,002	55.9 (5.2)	170 (8)	75.5 (13.8)	26.2 (4.0)	79%
Chingford	1,003	54.2 (6.0)	162 (6)	66.9 (11.8)	25.6 (4.3)	100%
FORCe	237	27.2 (5.9)	178 (9)	78.5 (12.8)	24.6 (3.1)	22%
JoCoOA	2,228	60.5 (9.0)	166 (9)	81.7 (18.1)	29.7 (6.0)	65%
MOST	3,026	62.5 (8.1)	169 (12)	87.9 (18.9)	30.7 (6.0)	60%
OAI	4,796	61.2 (9.2)	165 (24)	81.2 (16.4)	28.6 (4.8)	58%
RS	14,926	66.0 (10.5)	168 (10)	75.9 (13.9)	26.9 (4.1)	59%
SOF	9,704	71.6 (5.2)	159 (6)	67.0 (12.0)	26.4 (4.5)	100%
TASOAC	1,099	63.0 (7.5)	167 (9)	77.9 (15.0)	27.9 (4.8)	51%
**World COACH consortium**	**38,021**	**65.3 (8.6)**	**165 (12)**	**75.4 (14.5)**	**27.4 (4.6)**	**71%**

SD = Standard deviation; BMI = body mass index

For peer review only

Figure 1: PRISMA flow diagram detailing the literature search, screening, and inclusion process for the World COACH consortium.

PRISMA, Preferred Reporting Items for Systematic Reviews and Meta-Analyses; World COACH, Worldwide Collaboration on OsteoArthritis prediCtion for the Hip.

Figure 2: A simplified schema of the relational database used in the World COACH consortium.

World COACH, Worldwide Collaboration on OsteoArthritis prediCtion for the Hip.

For peer review only

References

1. Hunter DJ, Bierma-Zeinstra S. Osteoarthritis. *Lancet* 2019;393(10182):1745-1759. PMID: 31034380 doi: 10.1016/s0140-6736(19)30417-9.
2. GBD 2015 Disease and Injury Incidence and Prevalence Collaborators. Global, regional, and national incidence, prevalence, and years lived with disability for 310 diseases and injuries, 1990-2015: a systematic analysis for the Global Burden of Disease Study 2015. *Lancet* 2016;388(10053):1545-1602. PMID: 27733282 doi: 10.1016/s0140-6736(16)31678-6.
3. Gupta S, Hawker GA, Laporte A, et al. The economic burden of disabling hip and knee osteoarthritis (OA) from the perspective of individuals living with this condition. *Rheumatology (Oxford, England)* 2005;44(12):1531-1537. PMID: 16091394 doi: 10.1093/rheumatology/kei049.
4. Hunter DJ, Schofield D, Callander E. The individual and socioeconomic impact of osteoarthritis. *Nature Reviews Rheumatology* 2014;10(7):437-441. PMID: 24662640 doi: 10.1038/nrrheum.2014.44.
5. Clynes MA, Jameson KA, Edwards MH, et al. Impact of osteoarthritis on activities of daily living: does joint site matter? *Aging Clin Exp Res* 2019;31(8):1049-1056. PMID: 30903599 doi: 10.1007/s40520-019-01163-0.
6. Katz JN, Arant KR, Loeser RF. Diagnosis and Treatment of Hip and Knee Osteoarthritis: A Review. *JAMA* 2021;325(6):568-578. PMID: 33560326.
7. Bannuru RR, Osani MC, Vaysbrot EE, et al. OARSI guidelines for the non-surgical management of knee, hip, and polyarticular osteoarthritis. *Osteoarthritis Cartilage* 2019;27(11):1578-1589. PMID: 31278997 doi: 10.1016/j.joca.2019.06.011.
8. Rodriguez-Fontenla C, Calaza M, Evangelou E, et al. Assessment of osteoarthritis candidate genes in a meta-analysis of nine genome-wide association studies. *Arthritis Rheumatol* 2014;66(4):940-949. PMID: 24757145 doi: 10.1002/art.38300.
9. Jordan JM, Helmick CG, Renner JB, et al. Prevalence of hip symptoms and radiographic and symptomatic hip osteoarthritis in African Americans and Caucasians: the Johnston County Osteoarthritis Project. *J Rheumatol* 2009;36(4):809-815. PMID: 19286855.
10. Casartelli NC, Maffiuletti NA, Valenzuela PL, et al. Is hip morphology a risk factor for developing hip osteoarthritis? A systematic review with meta-analysis. *Osteoarthritis Cartilage* 2021;29(9):1252-1264. PMID: 34171473 doi: S1063-4584(21)00813-X [pii]
10.1016/j.joca.2021.06.007.
11. van Buuren MMA, Arden NK, Bierma-Zeinstra SMA, et al. Statistical shape modeling of the hip and the association with hip osteoarthritis: a systematic review. *Osteoarthritis Cartilage* 2021;29(5):607-618. PMID: 33338641 doi: 10.1016/j.joca.2020.12.003.
12. Doiron D, Burton P, Marcon Y, et al. Data harmonization and federated analysis of population-based studies: the BioSHaRE project. *Emerging Themes in Epidemiology* 2013;10(1):7622-7610-7612. PMID: 24257327 doi: 10.1186/1742-7622-10-12.

For peer review only

13. Crossley KM, Pandy MG, Majumdar S, et al. Femoroacetabular impingement and hip OsteoaRthritis Cohort (FORCe): protocol for a prospective study. *Journal of Physiotherapy* 2018;64(1):55. PMID: 29289588 doi: S1836-9553(17)30136-4 [pii]
10.1016/j.jphys.2017.10.004.
14. Cummings SR, Black DM, Nevitt MC, et al. Appendicular bone density and age predict hip fracture in women. The Study of Osteoporotic Fractures Research Group. *JAMA* 1990;263(5):665-668. PMID: 2404146.
15. Hart DJ, Spector TD. Cigarette smoking and risk of osteoarthritis in women in the general population: the Chingford study. *Ann Rheum Dis* 1993;52(2):93-96. PMID: 8447703 doi: 10.1136/ard.52.2.93.
16. Ikram MA, Brusselle GGO, Murad SD, et al. The Rotterdam Study: 2018 update on objectives, design and main results. *European Journal of Epidemiology* 2017;32(9):807-850. PMID: 29064009 doi: 10.1007/s10654-017-0321-4.
17. Laslett LL, Quinn SJ, Winzenberg TM, et al. A prospective study of the impact of musculoskeletal pain and radiographic osteoarthritis on health related quality of life in community dwelling older people. *BMC Musculoskelet Disord* 2012;13(Journal Article):2474-2413-2168. PMID: 22954354 doi: 10.1186/1471-2474-13-168.
18. Lester G. Clinical research in OA--the NIH Osteoarthritis Initiative. *J Musculoskelet Neuronal Interact* 2008;8(4):313-314. PMID: 19147953.
19. Nelson AE, Hu D, Arbeeva L, et al. Point prevalence of Hip Symptoms, Radiographic, And Symptomatic OA at Five Time Points: The Johnston County Osteoarthritis Project, 1991-2018. *Osteoarthritis and Cartilage Open* 2022;4(2) PMID: 36118130 doi: 100251 [pii]
10.1016/j.ocarto.2022.100251.
20. Segal NA, Nevitt MC, Gross KD, et al. The Multicenter Osteoarthritis Study: opportunities for rehabilitation research. *PM & R : the journal of injury, function, and rehabilitation* 2013;5(8):647-654. PMID: 23953013 doi: 10.1016/j.pmrj.2013.04.014.
21. Wesseling J, Boers M, Viergever MA, et al. Cohort Profile: Cohort Hip and Cohort Knee (CHECK) study. *International Journal of Epidemiology* 2016;45(1):36-44. PMID: 25172137 doi: 10.1093/ije/dyu177.
22. Iidaka T, Horii C, Muraki S, et al. Trends in prevalence of hip osteoarthritis over a 10-year period in Japan: The ROAD study 2005-2015. *Osteoarthr Cartil Open* 2022;4(3):100285. PMID: 36474937.
23. Nicholls AS, Kiran A, Pollard TCB, et al. The association between hip morphology parameters and nineteen-year risk of end-stage osteoarthritis of the hip: A nested case-control study. *Arthritis Rheum* 2011;63(11):3392-3400. doi: 10.1002/art.30523.
24. Jordan JM, Linder GF, Renner JB, et al. The impact of arthritis in rural populations. *Arthritis Care and Research* 1995;8(4):242-250. PMID: 8605262 doi: 10.1002/art.1790080407.
25. Nevitt MC, Felson DT, Lester G. The Osteoarthritis Initiative. Protocol for the cohort study. ;V 1.1 6.21.06. *National Institute of Arthritis, Musculoskeletal and Skin Diseases*.
26. Ikram MA, Brusselle G, Ghanbari M, et al. Objectives, design and main findings until 2020 from the Rotterdam Study. *Eur J Epidemiol* 2020;35(5):483-517. PMID: 32367290.

For peer review only

27. Ahedi H, Aitken D, Blizzard L, et al. Quantification of hip effusion-synovitis and its cross-sectional and longitudinal associations with hip pain, MRI findings and early radiographic hip OA. *BMC Musculoskelet Disord* 2020;21(1):020-03532-03537. PMID: 32778082 doi: 10.1186/s12891-020-03532-7.
28. Fortier I, Raina P, Van den Heuvel ER, et al. Maelstrom Research guidelines for rigorous retrospective data harmonization. *International Journal of Epidemiology* 2017;46(1):103-105. PMID: 27272186 doi: 10.1093/ije/dyw075.
29. Stang PE, Ryan Pb Fau - Racoosin JA, Racoosin Ja Fau - Overhage JM, et al. Advancing the science for active surveillance: rationale and design for the Observational Medical Outcomes Partnership. *Annals of Internal Medicine* 2010;153(1539-3704 (Electronic)):600-606. PMID: 21041580 doi: 153/9/600 [pii]
10.7326/0003-4819-153-9-201011020-00010.
30. Overhage JM, Ryan PB, Reich CG, et al. Validation of a common data model for active safety surveillance research. *Journal of the American Medical Informatics Association* 2012;19(1):54-60. PMID: 22037893 doi: amiajnl-2011-000376 [pii]
10.1136/amiajnl-2011-000376.
31. Altman RD, Bloch DA, Dougados M, et al. Measurement of structural progression in osteoarthritis of the hip: the Barcelona consensus group. *Osteoarthritis Cartilage* 2004;12(7):515-524. PMID: 15219566 doi: 10.1016/j.joca.2004.04.004.
32. Gold GE, Cicuttini F, Crema MD, et al. OARSI Clinical Trials Recommendations: Hip imaging in clinical trials in osteoarthritis. *Osteoarthritis Cartilage* 2015;23(5):716-731. PMID: 25952344 doi: 10.1016/j.joca.2015.03.004.
33. Kellgren JH, Lawrence JS. Radiological assessment of osteo-arthrosis. *Ann Rheum Dis* 1957;16(4):494-502. PMID: 13498604 doi: 10.1136/ard.16.4.494.
34. Macri EM, Runhaar J, Damen J, et al. Kellgren/Lawrence Grading in Cohort Studies: Methodological Update and Implications Illustrated Using Data From a Dutch Hip and Knee Cohort. *Arthritis Care and Research* 2022;74(7):1179-1187. PMID: 33450140 doi: 10.1002/acr.24563.
35. Nevitt MC, Lane NE, Scott JC, et al. Radiographic osteoarthritis of the hip and bone mineral density. *Arthritis Rheum* 1995;38(7):907-916. PMID: 7612040 doi: 10.1002/art.1780380706.
36. Croft P, Cooper C, Wickham C, et al. Defining osteoarthritis of the hip for epidemiologic studies. *American Journal of Epidemiology* 1990;132(3):514-522. PMID: 2389755 doi: 10.1093/oxfordjournals.aje.a115687.
37. Lane NE, Nevitt MC, Hochberg MC, et al. Progression of radiographic hip osteoarthritis over eight years in a community sample of elderly white women. *Arthritis Rheum* 2004;50(5):1477-1486. PMID: 15146417 doi: 10.1002/art.20213.
38. Altman RD, Gold GE. Atlas of individual radiographic features in osteoarthritis, revised. *Osteoarthritis Cartilage* 2007;15:A1-A56. PMID: 17320422 doi: S1063-4584(06)00328-1 [pii]
10.1016/j.joca.2006.11.009.
39. Wilkinson MD, Dumontier M, Aalbersberg IJ, et al. The FAIR Guiding Principles for scientific data management and stewardship. *Sci Data* 2016;3:160018. PMID: 26978244.

For peer review only

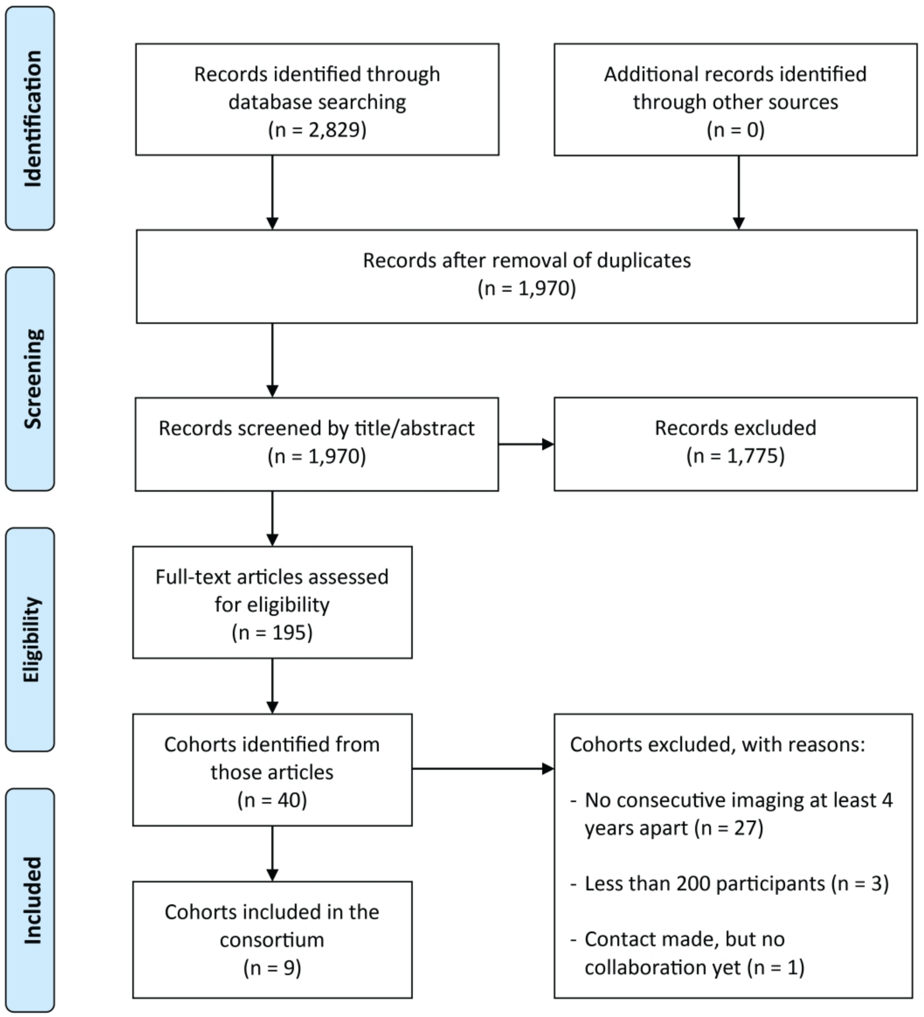

Figure 1: PRISMA flow diagram detailing the literature search, screening, and inclusion process for the World COACH consortium.

PRISMA, Preferred Reporting Items for Systematic Reviews and Meta-Analyses; World COACH, Worldwide Collaboration on OsteoArthritis prediCtion for the Hip.

434x474mm (236 x 236 DPI)

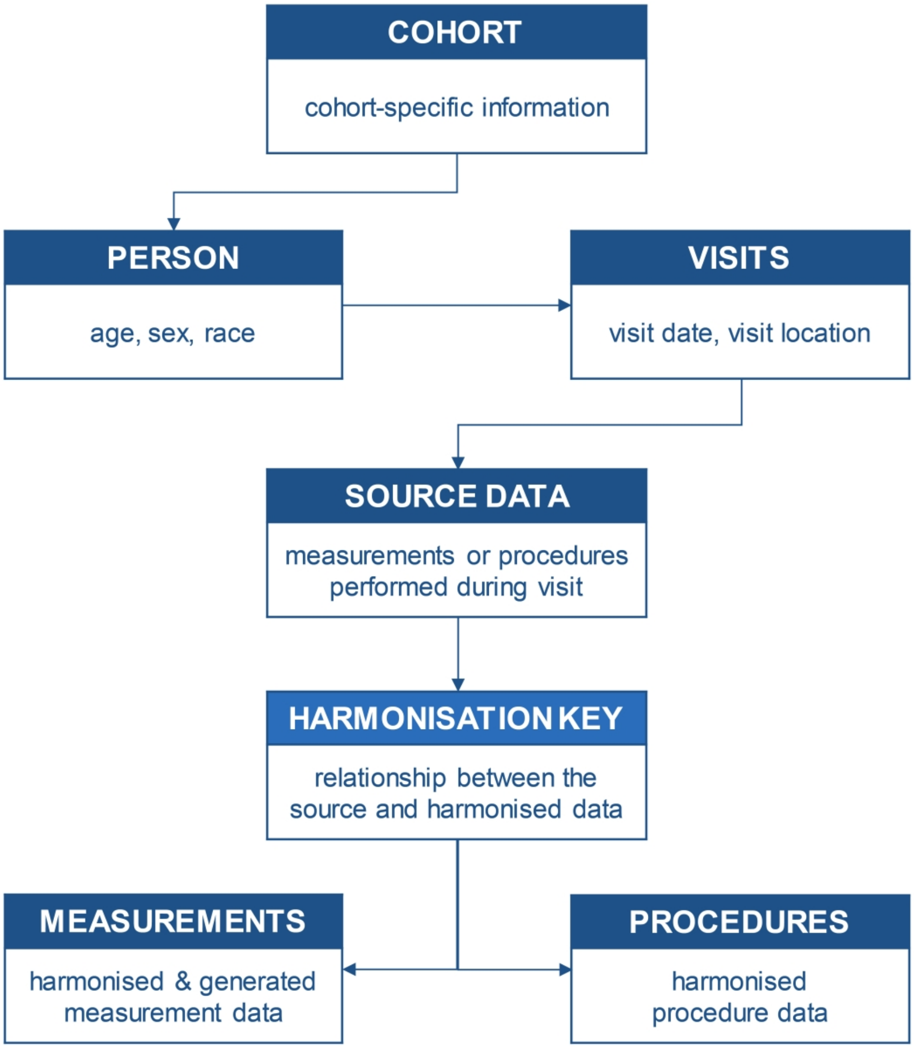

Figure 2: A simplified schema of the relational database used in the World COACH consortium.

World COACH, Worldwide Collaboration on OsteoArthritis prediCtion for the Hip.

422x480mm (236 x 236 DPI)

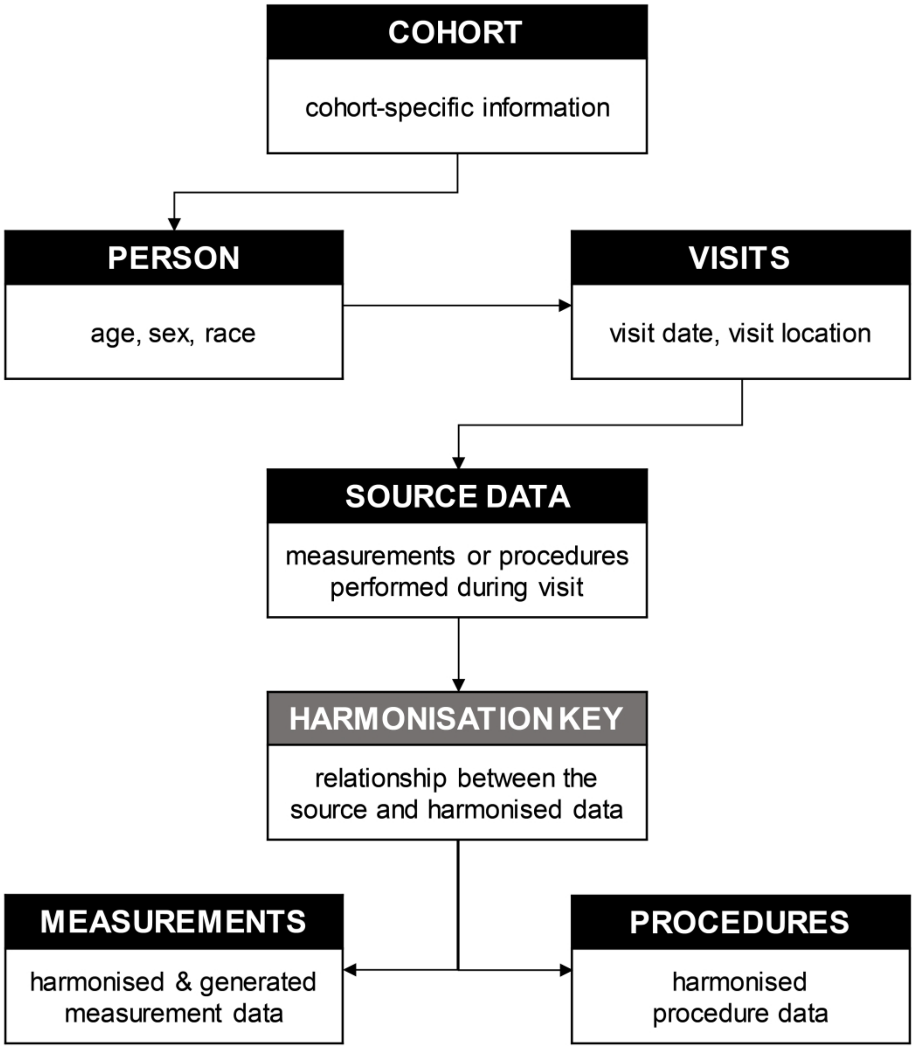

Caption : Figure 2: A simplified schema of the relational database used in the World COACH consortium.

World COACH, Worldwide Collaboration on OsteoArthritis prediCtion for the Hip.

422x480mm (236 x 236 DPI)

BMJ Open

Cohort profile: Worldwide Collaboration on OsteoArthritis prediCtion for the Hip (World COACH); an international consortium of prospective cohort studies with individual participant data on hip osteoarthritis.

Journal:	*BMJ Open*
Manuscript ID	bmjopen-2023-077907.R1
Article Type:	Cohort profile
Date Submitted by the Author:	15-Dec-2023

Complete List of Authors:	van Buuren, Michiel; Erasmus Medical Center, Department of Orthopaedics and Sports Medicine<br>Riedstra, Noortje; Erasmus Medical Center, Department of Orthopaedics and Sports Medicine<br>van den Berg, Myrthe; Erasmus Medical Center, Department of Orthopaedics and Sports Medicine<br>Boel, Fleur; Erasmus Medical Center, Department of Orthopaedics and Sports Medicine<br>Ahedi, Harbeer; University of Tasmania Menzies Institute for Medical Research<br>Arbabi, Vahid; UMC Utrecht, Department of Orthopedics; University of Birjand, Orthopaedic-Biomechanics Research Group, Department of Mechanical Engineering, Faculty of Engineering<br>Arden, Nigel; University of Oxford Nuffield Department of Orthopaedics Rheumatology and Musculoskeletal Sciences<br>Bierma-Zeinstra, Sita; Erasmus Medical Center, Department of General Practice<br>Boer, Cindy; Erasmus Medical Center, Department of Internal Medicine<br>Cicuttini, Flavia; Monash University, Department of Epidemiology and Preventative Medicine<br>Cootes, Timothy; The University of Manchester, Centre for Imaging Sciences<br>Crossley, Kay; La Trobe University School of Allied Health Human Services and Sport, La Trobe Sport and Exercise Medicine Research Centre<br>Felson, David; Boston University School of Medicine,<br>Gielis, Willem Paul; UMC Utrecht, Department of Orthopedics; UMC Utrecht, Department of Radiology<br>Heerey, Joshua; La Trobe University School of Allied Health Human Services and Sport, La Trobe Sport and Exercise Medicine Research Centre<br>Jones, Graeme; University of Tasmania Menzies Institute for Medical Research<br>Kluzek, Stefan ; University of Oxford Nuffield Department of Orthopaedics Rheumatology and Musculoskeletal Sciences<br>Lane, Nancy; University of California Davis School of Medicine, Department of Medicine<br>Lindner, Claudia; The University of Manchester, Centre for Imaging

	Sciences<br>Lynch, John A.; University of California San Francisco, Department of Epidemiology and Biostatistics<br>Van Meurs, J; Erasmus Medical Center, Department of Orthopaedics and Sports Medicine; Erasmus Medical Center, Department of Internal Medicine<br>Mosler, Andrea; La Trobe University School of Allied Health Human Services and Sport, La Trobe Sport and Exercise Medicine Research Centre<br>Nelson, Amanda ; The University of North Carolina at Chapel Hill, Thurston Arthritis Research Center<br>Nevitt, M; University of California San Francisco, Department of Epidemiology and Biostatistics<br>Oei, Edwin; Erasmus Medical Center, Department of Radiology & Nuclear Medicine<br>Runhaar, Jos; Erasmus Medical Center, Department of General Practice<br>Tang, Jinchi; Erasmus Medical Center, Department of Orthopaedics and Sports Medicine<br>Weinans, Harrie; UMC Utrecht, Department of Orthopedics; TU Delft, Department of Biomechanical Engineering<br>Agricola, Rintje; Erasmus Medical Center, Department of Orthopaedics and Sports Medicine
**Primary Subject Heading**:	Epidemiology
Secondary Subject Heading:	Radiology and imaging, Research methods, Rheumatology
Keywords:	Hip < ORTHOPAEDIC & TRAUMA SURGERY, RHEUMATOLOGY, RADIOLOGY & IMAGING, EPIDEMIOLOGY, GENETICS, Risk Factors

I, the Submitting Author has the right to grant and does grant on behalf of all authors of the Work (as defined in the below author licence), an exclusive licence and/or a non-exclusive licence for contributions from authors who are: i) UK Crown employees; ii) where BMJ has agreed a CC-BY licence shall apply, and/or iii) in accordance with the terms applicable for US Federal Government officers or employees acting as part of their official duties; on a worldwide, perpetual, irrevocable, royalty-free basis to BMJ Publishing Group Ltd ("BMJ") its licensees and where the relevant Journal is co-owned by BMJ to the co-owners of the Journal, to publish the Work in this journal and any other BMJ products and to exploit all rights, as set out in our licence.

The Submitting Author accepts and understands that any supply made under these terms is made by BMJ to the Submitting Author unless you are acting as an employee on behalf of your employer or a postgraduate student of an affiliated institution which is paying any applicable article publishing charge ("APC") for Open Access articles. Where the Submitting Author wishes to make the Work available on an Open Access basis (and intends to pay the relevant APC), the terms of reuse of such Open Access shall be governed by a Creative Commons licence – details of these licences and which Creative Commons licence will apply to this Work are set out in our licence referred to above.

Other than as permitted in any relevant BMJ Author's Self Archiving Policies, I confirm this Work has not been accepted for publication elsewhere, is not being considered for publication elsewhere and does not duplicate material already published. I confirm all authors consent to publication of this Work and authorise the granting of this licence.

For peer review only

Cohort profile: Worldwide Collaboration on OsteoArthritis prediCtion for the Hip (World COACH); an international consortium of prospective cohort studies with individual participant data on hip osteoarthritis.

M.M.A. van Buuren[1], N.S. Riedstra[1], M.A. van den Berg[1], F. Boel[1], H. Ahedi[2,3], V. Arbabi[4,5], N.K. Arden[6], S.M.A. Bierma-Zeinstra[1,7], C.G. Boer[8], F.M. Cicuttini[9], T.F. Cootes[10], K.M. Crossley[11], D.T. Felson[12], W.P. Gielis[4], J.J. Heerey[11], G. Jones[2], S. Kluzek[6,13], N.E. Lane[14], C. Lindner[10], J.A. Lynch[15], J.B.J. van Meurs[1,8], A. Mosler[11], A.E. Nelson[16], M.C. Nevitt[15], E.H.G. Oei[17], J. Runhaar[7], J. Tang[1], H. Weinans[4,18*], R. Agricola[1].

***Corresponding author**: H.H.weinans@umcutrecht.nl

Affiliations:

1. Department of Orthopaedics and Sports Medicine, Erasmus MC, University Medical Center Rotterdam, Rotterdam, The Netherlands.
2. Menzies Institute for Medical Research, University of Tasmania, Hobart, Tasmania, Australia.
3. Sydney Orthopaedic Research Institute, St Leonards, NSW, Australia .
4. Department of Orthopedics, University Medical Center Utrecht, Utrecht, The Netherlands.
5. Department of Mechanical Engineering, Faculty of Engineering, Orthopaedic-Biomechanics Research Group, Birjand, Iran.
6. Nuffield Department of Orthopaedics, Rheumatology and Musculoskeletal Sciences, Botnar Research Centre, University of Oxford, Oxford, UK.
7. Department of General Practice, Erasmus MC, University Medical Center Rotterdam, Rotterdam, The Netherlands.

For peer review only

8. Department of Internal Medicine, Erasmus MC, University Medical Center Rotterdam, Rotterdam, The Netherlands.
9. Department of Epidemiology and Preventive Medicine, Monash University, Melbourne, Victoria, Australia.
10. Centre for Imaging Sciences, The University of Manchester, Manchester, UK.
11. La Trobe Sport and Exercise Medicine Research Centre, School of Allied Health, Human Services and Sport, La Trobe University, Melbourne, Victoria, Australia.
12. Section of Rheumatology, Boston University School of Medicine, Boston, MA, USA.
13. NIHR Biomedical Research Centre, University of Nottingham, Nottingham, UK.
14. Department of Medicine, School of Medicine, University of California at Davis, Sacramento, CA, USA.
15. Department of Epidemiology and Biostatistics, University of California, San Francisco, CA, USA.
16. Thurston Arthritis Research Center, University of North Carolina at Chapel Hill, Chapel Hill, NC, USA.
17. Department of Radiology & Nuclear Medicine, Erasmus MC, University Medical Center Rotterdam, The Netherlands.
18. Department of Biomechanical Engineering, Delft University of Technology (TU Delft), Delft, The Netherlands.

For peer review only

Abstract

Purpose: Hip osteoarthritis (OA) is a major cause of pain and disability worldwide. Lack of effective therapies may reflect poor knowledge on its aetiology and risk factors, and result in management of end-stage hip OA with costly joint replacement. The Worldwide Collaboration on OsteoArthritis prediCtion for the Hip (World COACH) consortium was established to pool and harmonise individual participant data from prospective cohort studies. The consortium aims to better understand determinants and risk factors for the development and progression of hip OA, to optimise and automate methods for (imaging) analysis, and to develop a personalised prediction model for hip OA.

Participants: World COACH aimed to include participants of prospective cohort studies with ≥200 participants, that have hip imaging data available from at least 2 time points at least four years apart. All individual participant data, including clinical data, imaging (data), biochemical markers, questionnaires, and genetic data, were collected and pooled into a single, individual-level database.

Findings to date: World COACH currently consists of nine cohorts, with 38,021 participants aged 18 to 80 years at baseline. Overall, 71% of the participants were female and mean baseline age was 65.3 ± 8.6 years. Over 34,000 participants had baseline pelvic radiographs available, and over 22,000 had an additional pelvic radiograph after 8–12 years of follow-up. Even longer radiographic follow-up (15–25 years) is available for over 6,000 of these participants.

Future plans: The World COACH consortium offers unique opportunities for studies on the relationship between determinants/risk factors and the development or progression of hip OA, by using harmonised data on clinical findings, imaging, biomarkers, genetics, and

For peer review only

lifestyle. This provides a unique opportunity to develop a personalised hip OA risk prediction model and to optimise methods for imaging analysis of the hip.

Keywords: Hip osteoarthritis, hip morphology, genetics, prediction, epidemiology, risk factors

Strengths and limitations of this study

- The Worldwide Collaboration on OsteoArthritis prediCtion for the Hip (World COACH) consortium brings patients together with a highly qualified and multidisciplinary team of experts and young investigators in the field of hip osteoarthritis, with backgrounds in orthopaedic surgery, rheumatology, physical therapy, general practice, genetics, epidemiology, biostatistics, technical medicine, biomechanical engineering, radiology, imaging science, and artificial intelligence.
- The World COACH consortium is unique for having harmonised individual participant data on clinical measurements, radiological imaging, biochemical markers, lifestyle and diet, comorbidities, medication, physical and cognitive functioning, quality of life and genetics from over 38,000 people, both from the general population as well as from specific populations at risk for hip osteoarthritis.
- The World COACH consortium has sequential hip radiography available for each participant with a follow-up duration ranging from 5 to over 25 years.
- The main limitations of the consortium are the geographic origins of the included cohorts (Western world) and the heterogeneity in collected data by the cohorts, which may limit the possibilities of harmonisation.

For peer review only

Introduction

Osteoarthritis (OA) is a common disease and a leading cause of disability in adults(1). Over 500 million people are affected by OA worldwide, leading to a global prevalence of around 7%(2). The forecast for OA is alarming; with an ageing population, the prevalence is expected to rise dramatically in the coming decades. The direct healthcare costs of OA in various high-income countries account for 1% - 2.5% of the gross domestic product(3, 4). OA can affect any joint and is most prevalent in the knee and hip, where it also leads to the greatest physical disability(5). Due to a subsequent decrease in physical activity, hip OA also leads to more comorbidities and a higher age-adjusted mortality(6).

Despite the tremendous burden of hip OA, there is no cure available. Therefore, current strategies focus on symptomatic treatment with only a modest effect(7). This may partly result from a lack of knowledge on the aetiology, pathophysiology and risk factors of hip OA. Hip OA is a heterogeneous disease in which the risk factors and aetiology can differ widely from patient to patient. In contrast to knee OA, few large studies have focused on hip OA risk prediction so far. Up until 2022, 31 multivariable prediction models for incident knee OA have been published, while only four exist for hip OA. On top of that, all four have been created with data from Dutch cohort studies only (8). This accentuates the need for more international collaborations in hip OA research.

Additionally, this lack of knowledge regarding person-specific risk factors for hip OA makes efficient and effective preventative and treatment strategies challenging, if not impossible, thus only one-size-fits-all treatment options for hip OA are available to date. Still, some risk

factors for hip OA have been identified on a group level, such as age, gender(5), obesity(9), genetics(10), race(11), and hip morphology (such as cam and pincer morphology or acetabular dysplasia)(12, 13). However, each risk factor has only weak or even conflicting associations with hip OA, they have mainly been studied in single, heterogeneous cohorts, and are typically studied separately from each other. These single studies are underpowered to predict the risk of hip OA on an individual level.

Next to allowing for risk prediction on an individual level, a large dataset also allows for applying techniques from the rapidly emerging field of radiological image processing. These techniques could be used for classification of hip OA or diagnosing hip morphology. This does raise an additional question: how should we make optimal use of these techniques, both in a research setting and in clinical practice? Research on the use of artificial intelligence (AI) in hip OA, including the use of machine learning and deep learning, has so far been done in single cohort studies only(14, 15). This may limit the generalisability of the results and the continuation of the research into real-wold applications.

To overcome these challenges, we believe that the prospective cohort study design is ideal to better understand which individuals are at risk of developing hip OA and of progressing to end-stage disease. Harmonising data from multiple cohort studies into an individual participant-level database provides a large sample size, which may allow for individualised or at least subgroup-specific risk estimates. Further, large sample sizes and diverse cohorts from all over the world improve the generalisability of the findings(16). To meet this need,

For peer review only - http://bmjopen.bmj.com/site/about/guidelines.xhtml

139 the Worldwide Collaboration on OsteoArthritis prediCtion for the Hip (World COACH) consortium was initiated in 2018. The consortium aims to better understand risk factors for the development or progression of hip osteoarthritis, and to optimise and automate methods for analysing radiological images of the hip. This will be pursued through studying multiple research questions within the consortium.

CONSORTIUM DESCRIPTION

Objectives and research questions

To study the research questions of the World COACH consortium, the consortium currently has five separate work packages. The first work package is **Methodology**, of which the goal is to discover, optimise, automate and validate new methods in OA research, such as an automated pipeline for hip morphological analyses and developing algorithms for the detection of radiographic hip OA (RHOA). This includes the application of AI. **Hip Morphology** is the second work package and it focuses on investigating associations between hip morphology and hip OA. Known morphological risk factors such as acetabular dysplasia, pincer morphology, and cam morphology will be investigated, as well as general hip shape captured with statistical shape modelling. The third work package is **Genetics**, of which the aim is to study associations between genetics, hip morphology, environmental factors, and OA, by applying Genome-Wide Associations Studies (GWAS) among other methods. The fourth work package is **Clinical Measures**, comprising physical examinations, questionnaires, quality of life and blood and urine samples. The aim is to study the associations between these measures and the development of hip OA. Finally, the

fifth work package (**Prediction Modelling**) combines data and results from all other work packages to develop a personalised risk prediction model for the development and progression of hip OA, using both conventional and AI-driven methods.

Cohort inclusion and consortium establishment

Prospective cohort studies were considered eligible if they had hip radiography – and optionally computed tomography (CT) and/or magnetic resonance imaging (MRI) – available at two or more points in time, at least four years apart, and if they had a minimum of 200 participants at baseline. These criteria were applied at cohort level, but not participant level, thus having some participants with missing radiographs was not a reason to exclude a cohort. A systematic literature search was conducted in Embase, Ovid MEDLINE and Cochrane CENTRAL to identify all studies that fulfilled the inclusion criteria. The search was first carried out in 2017 and was repeated in October 2020 and again in March 2023. Titles and abstracts were screened independently by two researchers (MvB and RA), and all described cohorts were further investigated, both by reading the full texts of the screened references and by additional internet searches. A PRISMA flow diagram of the search and inclusion process is presented in **Figure 1**. In summary, we screened a total of 1,970 records by title and abstract, of which we assessed 195 records in detail. We identified 40 study cohorts, 10 of which we considered eligible for the consortium. Investigators from the eligible cohorts were contacted and asked to collaborate. To date, 9 cohorts have been included in the consortium(17-25), and contact has been initiated with the remaining eligible cohort(26). The systematic search will be repeated every 2 years to identify newly eligible cohorts.

For peer review only

After the first search, the initiator of the World COACH consortium (RA) contacted the principal investigators of the 9 identified cohorts to discuss the consortium's aims. A live meeting with principal investigators of 8 cohorts, as well as other individuals interested in participating in the consortium, was held during the OsteoArthritis Research Society International (OARSI) world congress in Liverpool, 2018. During this meeting, the overall aims of the consortium were presented, and an inventory of the support for initiating the consortium was assessed. With unanimous support for the consortium, we decided to establish this initiative. Legal agreements for data sharing were drafted and executed. Since 2018, quarterly meetings have been held with the collaborators to determine the aims and research plans of the consortium.

Description of the cohorts

A summary of the included cohorts can be found in **Table 1**. All included studies are prospective cohort studies with a minimum of 5 and a maximum of more than 25 years of follow-up. Some cohorts still have ongoing data collection(17, 20, 24). The earliest data collection in any cohort started in 1986(18), and the most recent baseline data collection started in 2016(20). Most included cohorts are population-based studies. Exceptions are the CHECK cohort, in which participants had hip or knee complaints, MOST and OAI, which both included individuals with or at high risk of knee OA, and FORCe, which included participants with hip and/or groin pain. At least 6 cohorts included non-white individuals, and 6 cohorts studied both men and women. The total World COACH population includes participants from 3 continents. Across cohorts, participants were aged 18 to 80 years at

For peer review only

enrolment. The aims and methods of each individual cohort are shortly described below, emphasising those characteristics that correspond with the consortium's inclusion criteria (e.g. radiographic protocols are highlighted, but not CT or MRI).

Cohort Hip and Cohort Knee (CHECK) study

The CHECK study was a multi-centre prospective cohort study in the Netherlands that ran from 2002 until 2015 for a total of 10 years follow-up(25). The aim was to study the course, prognosis and underlying mechanisms of early symptomatic OA. The study included 1,002 participants aged 45–65 years, with a first episode of pain in the hip and/or knee. Participants were eligible if they had not yet visited a general practitioner (GP) or were within 6 months of their first visit to the GP for these symptoms, or if they had never visited a GP before for these symptoms, and if there was no other diagnosis that could explain the symptoms at the time of inclusion. Participants were recruited between October 2002 and September 2005, mostly through local newspaper articles and advertisements, and the website of the Dutch Arthritis Society (69% of inclusions). Additionally, eligible individuals were referred by their GP to one of 10 participating general and university hospitals (6%), recruited through a flyer, family member or a friend (12%), and for the remainder it was not recorded. Standardised weight-bearing anteroposterior (AP) hip or pelvic radiographs, using a wedge to get the hips in 15° internal rotation, were obtained at baseline and at 2, 5, 8 and 10 years of follow-up. At 10 years follow-up, 87% of the baseline cohort had RHOA scores completed.

The Chingford 1000 women study

The Chingford study was a population-based prospective cohort study that aimed to assess musculoskeletal disease in the female population(19). It ran from 1989 to 2010, having over 20 years of follow-up. The study recruited asymptomatic female participants, aged 45–64 years, from the registry of a large general practice (over 11,000 patients) in Chingford, London, UK. All 1,353 women in that age range were invited to participate, of which 1,003 were included. Standardised supine AP pelvic radiographs, using a small sand bag under the knees to minimise hip rotation, were obtained at year 2, 8 and 20 of follow-up(27). After 8 years, 99% of the participants who had baseline RHOA scores, also had a follow-up score. At 15 years, 77% of the original cohort were still being followed up.

Femoroacetabular impingement and hip OsteoaRthritis Cohort (FORCe) study

The FORCe study is an ongoing prospective cohort study aiming to evaluate changes in hip joint structure in sub-elite soccer and Australian football players with hip and/or groin pain, with a focus on early hip OA features(17). Participants were recruited between August 2015 and October 2018. The study included 239 participants, aged 18-50 years, who were recruited through advertisements at sporting venues and from orthopaedic, sports medicine or physical therapy clinics. Participants were eligible if they had self-reported hip and/or groin pain for >6 months, with a gradual onset, and pain with a score between 3 and 8 on an 11-points numerical rating scale (NRS). They also had to have a positive flexion-adduction-internal-rotation (FADIR) test in at least one hip, indicative of femoroacetabular impingement. At baseline, all participants underwent standardised supine AP pelvic radiographs with the feet in 15° internal rotation using a positioning aid,

and MRI of the hips. The study is currently inviting participants for a 5-year follow-up visit, that comprises pelvic radiography and MRI according to the same standardised protocols.

Johnston County Osteoarthritis Project (JoCoOA)

The JoCoOA was a population-based cohort study with up to 30 years of follow-up(23, 28). Its aim has been to examine the incidence, prevalence and progression of osteoarthritis in Black and White men and women in a rural county. The study started in 1991 and data collection ended in 2018. Participants, all non-institutionalised black and white men and women, were drawn by probability sampling from the population of Johnston County, North Carolina, USA. The study included 4,337 participants aged ≥45 years. Standardised supine AP pelvic radiographs with the feet in 15° internal rotation were obtained at baseline, and then every 5–6 years, except for women under the age of 50 at the time of assessment (per protocol). Follow-up rates over the years have been between 50-60% for each subsequent visit, with the main reason for loss to follow-up being death (around 17% each visit).

Multicenter Osteoarthritis Study (MOST)

MOST is a multicentre prospective cohort study in the USA that started in 2003 and has followed participants for 20 years so far (24). The aim was to study risk factors for the development and progression of knee osteoarthritis and knee pain. Two centres in Birmingham (Alabama) and Iowa City (Iowa) recruited participants with pre-existing knee OA or those at high risk for knee OA from the general population. Eligible individuals were identified through databases from health insurance companies, voter registration tapes,

commercial list brokers, and other sources, after which they were sent invitation letters and study brochures. The study included 3,026 individuals aged 50–79 years in its initial phase, with a new cohort of 1,500 individuals included in 2016-2018. A standardised weight-bearing AP full-limb radiograph of the lower extremities (including the pelvis) with the tibial tubercles facing forward and the X-ray beam centred at the knee was obtained at baseline, and again at 5 years of follow-up. Because the pelvis was included in these sequential full-limb radiographs, this cohort study on knee OA was also eligible for inclusion in the consortium. After 5 years, 99% of participants that had baseline RHOA scores, also had follow-up scores completed.

Osteoarthritis Initiative (OAI)

The OAI study was a multicentre prospective cohort study of knee OA in the USA(22, 29). OAI aimed to provide resources to enable a better understanding of prevention and treatment of knee OA. It was initiated in 2002 and the entire cohort finished its 8-year follow-up in 2015, but the follow-up continues for certain subsets of participants. The OAI study has included 4,796 participants with pre-existing knee OA or those at high risk for developing knee OA, from the general populations of Baltimore (Maryland), Columbus (Ohio), Pittsburgh (Pennsylvania), and Pawtucket (Rhode Island). Participants were aged 45–79 years at enrolment. These participants were contacted through focused mailings, advertisements in local newspapers, presentations at church, community or civic meetings, and a website about knee pain and osteoarthritis. Standardised weight-bearing AP pelvic radiographs using a v-shaped foot-positioning frame to get the feet in 5° of internal rotation were obtained at baseline, 4 years, and 8 years follow-up. The inclusion of pelvic

radiography made this knee OA study also eligible for the consortium. RHOA scores were available for 77% at the 4-year follow-up visit, while the 8-year radiographs have yet to be scored for hip OA.

The Rotterdam Study (RS)

The RS is an ongoing prospective population-based cohort study in a district of the city of Rotterdam, the Netherlands(30). It aims to address determinants and occurrence of cardiovascular, neurological, musculoskeletal, ophthalmologic, psychiatric, and endocrine diseases in the elderly. After the pilot in 1989, the study started recruiting in 1990, and it currently has over 25 years of follow-up. The names and addresses of eligible participants were drawn from the municipal register, after which random clusters of potential participants got invited through a letter sent to their home, followed up by a phone call. Up to 2008, the study had included 14,926 participants (72% of 20,744 invitees) aged ≥45 years, divided into three sub-cohorts from different enrolment periods, namely RS-I, RS-II and RS-III. Recruitment of a fourth sub-cohort (RS-IV) started in 2016 and has recently been finished. Data from RS-IV will also be included once they fulfil the inclusion criteria. Standardised weight-bearing AP pelvic radiographs with the feet in 10˚ internal rotation were obtained at baseline, and then approximately every 4–6 years. Because of the different sub-cohorts with different follow-up schemes, there is no single follow-up rate. The follow-up rate decreases over time, especially after 12 years and over, as can be expected in an ageing population.

Study of Osteoporotic Fractures (SOF)

SOF was a multi-centre prospective population-based cohort study of community-dwelling women aged ≥65 years(18). The primary purpose of SOF was to describe risk factors for osteoporotic fractures. Women were recruited between September 1986 and October 1988 from 4 metropolitan areas in the USA: Baltimore (Maryland), Pittsburgh (Pennsylvania), Minneapolis (Minnesota) and Portland (Oregon). Eligible women were identified in multiple ways: through membership lists from health insurance companies, jury selection lists, voter registration lists, and drivers' licenses and identification cards lists. Women received a letter and brochure inviting them to participate. The original cohort included 9,704 mostly Caucasian women who had not undergone bilateral hip replacement and were able to walk without assistance. The cohort has over 20 years of prospective data about osteoporosis. Standardised supine AP pelvic radiographs with the hips in 15–30° internal rotation were obtained at baseline and after 8 years of follow-up. The follow-up rate for RHOA scores was 100%.

Tasmanian Older Adults Cohort (TASOAC) study

The TASOAC study is an ongoing prospective population-based cohort study of 1,099 community-dwelling men and women, aged 50–80 years(21). The study aimed to identify factors associated with the development and progression of OA in multiple joints, including the hip. Eligible participants were randomly selected from the electoral roll in Southern Tasmania, using sex-stratified simple random sampling without replacement (response rate 57%). Participants were excluded if they were institutionalised or if they reported a contraindication for MRI. Enrolment started in 2002 and the cohort had follow-up

moments at approximately 2.7 years, 5 years and 10 years. Standardised weight-bearing AP pelvic radiographs with the feet in 10° internal rotation were obtained at baseline and after 10 years of follow-up. A subgroup (n=250) had MRI of the right hip in the sagittal plane at 2.7 and 5 years follow-up(31). At inclusion, the TASOAC study did not yet have OA scores available for their 10-year follow-up. These will be added at a later time point.

DATA HARMONISATION

Retrospective harmonisation is an intricate process, considering few original studies have used identical collection methods and procedures. Our harmonisation process will be based both on expert opinion within the consortium, as well as on the Maelstrom Research guidelines for rigorous retrospective data harmonisation(32).

Defining the DataSchema

We started by analysing the present literature on the included studies (e.g. study protocols, published papers) to evaluate sources of study heterogeneity. The next step was to define variables and evaluate the harmonisation potential. All available variables from individual studies within the consortium were identified and systematically entered in a DataSchema(32), categorised in thirteen sections: demographic data, physical examinations and anthropometry, radiographic measurements of OA, questionnaires, family history, procedures, biospecimens, lifestyle and diet, comorbidities, medication, physical and cognitive functioning, quality of life, and genetics. This allowed us to evaluate comparability between studies. Next, all data was catalogued based on their characteristics. All similar variables that indicate the same measurement were grouped together and

renamed using a common pooled variable. Finally, the process of data harmonisation was initiated, for which we used and will continue to use one of the established approaches, depending on the data(32):

- **Simple calibration model**: will be used to transform continuous variables into new continuous variables (e.g. transforming height in inches to height in centimetres). The distribution of the values will be compared across cohorts to assess for differences within the measurement.
- **Algorithmic transformation**: will be used to harmonise continuous or categorical variables with combinable ranges or categories (e.g. race or ethnicity, education level).
- **Standardisation model**: will be used to harmonise the same constructs measured with different scales, when there are no bridging items available (e.g. two independent questionnaires on hip symptoms)
- **Latent variable model**: will be used to harmonise variables with different scales that have some bridging items (e.g. OA grade based on the Kellgren-Lawrence (KL), Croft or OARSI atlas classification, which all contain items such as joint space narrowing and osteophytes).

Data storage and processing

After establishing data transfer agreements with each included cohort, all required individual participant data were transferred to a central server. Variables were then prepared to be entered into a relational database using the Observational Medical Outcomes Partnership (OMOP) Common Data Model (CDM) structure(33, 34). The CDM is a

'person-centric' model and is optimised for observational research purposes such as identifying patient populations with certain outcomes (such as hip OA), characterisation of these populations for various parameters (including risk factors), and predicting the occurrence of the outcome in individuals. Variables were clustered in domains using the CDM's standardised vocabularies. Although the variables were mapped to the standardised vocabularies, we also stored the original source values, to ensure that all data entries can be traced when locating or preventing unforeseen errors. The OMOP CDM does not require specific software and can be realised in any relational database software. We currently use an advanced open-source relational database system (PostgreSQL version 15.2, PostgreSQL Global Development Group) which uses the SQL data definition and query language. The stored variables from each cohort and their individual participants include demographics and follow-up visits, along with measurements and procedures performed at each visit. This original data collection setup is used within the relational database model, which contains seven linked tables (**Figure 2**). The person table contains demographics of the included individual, such as biological sex, year of birth, and the originating cohort identification number, which links to the descriptive cohort table. The visit occurrence table contains the different time points and study sites (where available) at which data was collected from each individual. The measurement and procedure occurrence tables contain harmonised and newly generated World COACH variables for each specific follow-up visit (e.g. harmonised RHOA score based on available KL or modified Croft grades). The harmonisation steps are documented and harmonised values are linked to their source value through the harmonisation key table.

413 **Outcome measures**

414 The main outcome is the development of hip OA within the various follow-up periods,
415 although there are several outcomes of interest for secondary analyses. Hip OA could be
416 defined structurally by radiological indices, clinically by pain and/or functional indices, and
417 if possible, by a combination of these two. Radiographs are the only validated and
418 recommended imaging modality to investigate hip OA as a structural outcome(35, 36).
419 Pelvic radiographs have been read for the presence and severity of radiographic OA using
420 either the KL classification(37, 38), the (modified) Croft classification(39-41), or the atlas
421 of individual radiographic features in osteoarthritis (OARSI atlas)(42). The inclusion
422 criterion of having hip radiography available at two or more points in time, at least four
423 years apart, was set to determine the presence or absence of RHOA at both time points.
424 This is necessary to distinguish between incident RHOA (in case of no RHOA at baseline) or
425 progression of RHOA (in case of RHOA at baseline). Different pain scores, such as visual
426 analogue scales (VAS), Western Ontario and McMaster Universities Osteoarthritis Index
427 (WOMAC) pain scale, the Hip Disability and Osteoarthritis Outcome Score (HOOS), and
428 other scores will be harmonised into a single pain index if and where possible.

429

430 **FINDINGS TO DATE**

431 As of submission, the World COACH database contains data on 39,805 individuals. The
432 mean age of all World COACH participants at baseline was 65.4 ± 8.8 years and the study
433 sample consisted of 27,957 (70.2%) women. Mean BMI ranged from 24.6 kg/m^2 in the
434 FORCe study to 30.7 kg/m^2 in MOST, with an overall mean BMI of 27.5 kg/m^2 . The amount
435 of available baseline pelvic and/or hip radiographs is 34,257 (**Table 2**).

The amount of currently available RHOA scores in the included studies is shown in **Supplemental Table S1**, both at baseline and at each of the studies' respective follow-up visits. At baseline, there were almost 60,000 hips with a valid RHOA score available. At both the 4-year and the 5-year mark, 10,000 hips were scored, and around 20,000 hips have a score after 8 years of follow-up. The numbers of hips with a valid OA score logically decreases with longer follow-up times (10, 12, 15, 20 and 25 years of follow-up). The number of RHOA scores available to date may increase in future publications as we plan to score available radiographs that currently miss radiographic OA scores.

Currently available baseline RHOA scores are shown in **Table 3**. Most included cohorts used several methods for RHOA scoring such as KL, (modified Croft), and OARSI individual features. At baseline, 36,065 hips (61.1% of those with available RHOA scores) showed no signs of RHOA (score 0), and 17,778 hips (30.1%) had early or doubtful RHOA (score 1). Definite RHOA or a total hip replacement was present in 5,135 hips at baseline (8.7%). When looking at hips with both baseline and follow-up RHOA scores available, 42,619 hips were free of definite RHOA at baseline. Within this group, 3,207 (8%) of the hips developed incident RHOA at follow-up (**Supplemental Table S2**).

Other available variables of which the harmonisation process is still ongoing (besides those shown in the tables) are: ethnicity/race (all cohorts), socioeconomic status (all cohorts), smoking status (all cohorts), hip pain (CHECK, Chingford, FORCe, JoCoOA, MOST, RS, TASOAC), hip range of motion (CHECK, FORCe, JoCoOA), bone mineral density (Chingford,

For peer review only

OAI, SOF, RS, TASOAC), physical activity (CHECK, Chingford, FORCe, JoCoOA, MOST, OAI, RS, TASOAC).

STRENGTHS AND LIMITATIONS

The main strength of the World COACH consortium is its rich variety of harmonised, individual participant data from all available prospective cohort studies on hip OA worldwide. Although this offers significant challenges, it has the potential to improve the generalisability of our findings. The large sample size offers unique opportunities to study the relationship between different risk factors and the development and progression of hip OA on an individual level, as well as the identification of high-risk subgroups. It also allows for analysis of interactions between these factors, such as the effect of obesity across different hip shape variations. This will hopefully allow for the creation of the first person-specific and/or subgroup-specific risk estimation of developing hip OA. This personalised model can in turn be used to identify both high-risk individuals and the factors that contribute to this risk. In turn, this provides opportunities for future studies on prevention and individualised OA treatment. Furthermore, the World COACH consortium strives to offer solutions to some of the greatest epidemiological issues in terms of hip OA research by testing, automating, and validating methodological issues related to image analysis, with the potential of providing a benchmark for imaging analysis in hip OA research. Finally, the extensive dataset allows for investigating an array of secondary research questions along with the main aims of the consortium.

The strength of a relational database is that it is possible to enrich the existing consortium data with data from new cohorts once they meet the inclusion criteria, without the need to

For peer review only

482 restructure the datasets. The flexible structure of relational databases allows for seamless
483 expansion to handle increasing volumes of data and it can easily adapt to frequent updates
484 or deletions.
485 Limitations of the consortium include the limited geographic selection of cohorts from the
486 Western world (Australia, Europe and the United States). To date, no cohorts have been
487 included from Africa, Asia or South America, which may limit the generalisability of
488 findings. There is some heterogeneity in the populations from which World COACH
489 participants were originally drawn. Most cohorts have included participants from the
490 general population, albeit with an age restriction (Chingford, JoCo, the Rotterdam Study,
491 SOF and TASOAC), but some cohorts included participants with specific characteristics
492 (CHECK, FORCe, MOST, OAI). This may limit generalisability of the findings and is
493 something we have to account for in future analyses. We will consider the use of different
494 statistical methods that could account for cohort differences and address heterogeneity.
495 Most cohorts included participants aged 45 years or older, while only the FORCe cohort
496 included younger participants. Although people aged over 45 years represent the vast
497 majority of the hip OA population, we will be underpowered to externally validate findings
498 in people younger than 45 years. The World COACH consortium is also limited by the
499 heterogeneity in collection of variables by the cohorts, which was inherently done in
500 slightly different ways. This requires harmonisation of variables, which is mainly based on
501 (potentially subjective) expert-based criteria. On the other hand, pooling of the data creates
502 far greater statistical power than previously possible. Finally, although a subset of the data
503 consists of 3-dimensional imaging data such as CT or MRI, most analyses will be performed
504 using plain AP pelvic radiographs. As stated by the American College of Rheumatologists

For peer review only

(ACR, USA)(43), the National Institute for Health and Care Excellence (NICE, UK)(44), and the European Alliance of Associations for Rheumatology (EULAR, EU)(45), imaging is not necessary for the diagnosis of hip osteoarthritis in clinical practice. Still, radiographs probably contain valuable (hidden) predictive information for hip OA, and they are extensively used in daily clinical practice. Radiographs are also the only valid method to diagnose structural hip OA so far and are a simple and inexpensive tool for use in large clinical studies. Findings from this consortium may also guide primary care providers as to which patients should be sent for radiographic imaging, and which patients could start conservative treatment based on a clinical diagnosis of hip OA.

For peer review only

Collaboration

We will provide a harmonised database containing all prospective data on hip OA. We encourage the use of data by third parties, although this is subject to approval by the steering committees of the World COACH consortium and the participating cohorts, as well as to legal boundaries regarding data ownership. To streamline the processing of third-party requests, we have developed a standardised data request form that can be distributed and reviewed uniformly. This will ensure consistency in the way data requests are handled within World COACH.

Our approach to data storage involves the Findable, Accessible, Interoperable and Reusable (FAIR) principles(46). This will be achieved by using unique and persistent identifiers, by adhering to the Observational Health Data Sciences and Informatics (OHDSI) terminology where possible, and by implementing standardised access protocols to make data available upon request. By adhering to the FAIR principles, we aim to promote collaboration and

transparency to advance scientific research in the field of hip OA and beyond. The relational database supports data storage that is compatible with other data sources and formats, enabling seamless integration.

Finally, the project will be overseen by two committees: a steering committee and an advisory committee, which have quarterly meetings regarding the consortium. Both committees consist of a diverse team of experienced researchers and clinicians in the areas of osteoarthritis, rheumatology, epidemiology and image processing. Their combined expertise will provide valuable guidance and ensure the project's success.

More information on the consortium and on data requests can be obtained from the website: www.worldcoachconsortium.com.

Patient and public involvement

A patient and public committee (PPC) is being formed to ensure that the wider public is represented in World COACH. World COACH aims to ensure that all projects are relevant, meaningful and have impact on the people and patients it aims to serve. This includes not only patients with hip OA, but also families, caregivers and members of the general public. The PPC will be involved in prioritising research questions and in helping to shape the long-term vision of World COACH with particular consideration for the interests of the public and patients with hip OA. Our goal is to engage with a relevant population by promoting our project at various events. Additionally, we have made our research team accessible to the public through the World COACH website, where individuals can contact us directly via email, and through public meetings such as a local "OA cafe". We actively encourage such interactions in our presentations to foster engagement and promote

For peer review only

greater understanding of our research to the public, and for the team to better understand what is relevant and important to patients.

Acknowledgments

We would like to thank all participants of the cohort studies that are involved in the World COACH consortium. We gratefully acknowledge all international organisations that collaborated with the cohort studies in World COACH, as well as the Osteoarthritis Research Society International (OARSI) for endorsing the World COACH consortium. We thank the (non-profit) funding bodies who financially support the World COACH consortium: the Dutch Arthritis Society (grant no. 18-2-203 and 21-1-205), the Dutch Research Council (NWO Veni grant scheme no. 09150161910071) and the Erasmus MC, University Medical Center, Rotterdam (Erasmus MC Fellowship). For the purposes of open access, the authors have applied a CC BY public copyright licence to any Author Accepted Manuscript version arising from this submission.

CHECK: The CHECK study was initiated by the Dutch Arthritis Society and performed within: Erasmus Medical Center Rotterdam; Kennemer Gasthuis Haarlem; Leiden University Medical Center; Maastricht University Medical Center; Martini Hospital Groningen/Allied Health Care Center for Rheum. and Rehabilitation Groningen; Medical Spectrum Twente Enschede/Ziekenhuisgroep Twente Almelo; Reade, formerly Jan van Breemen Institute/VU Medical Center Amsterdam; St.Maartens-kliniek Nijmegen; University Medical Center Utrecht and Wilhelmina Hospital Assen.

Chingford: We would like to thank all the participants of the Chingford Women Study, Professor Nigel Arden, Professor Tim Spector, Dr Deborah Hart, Mr Gem Lawson, Maxine

For peer review only

Daniels and Alison Turner for their time and dedication and Arthritis Research UK for their funding support to the study and the Oxford NIHR Musculoskeletal Biomedical Research Unit for funding contributions.

FORCe: We would like to thank the staff at Imaging at Olympic Park and Qscan radiology clinics who continue to assist in study data collection, and the study participants. The FORCe study was funded by two National Health and Medical Research Council (NHMRC) of Australia project grants (GNT1088683 and GNT1156674).

JoCoOA: Support for data from the Johnston County Osteoarthritis Project was provided in part by: the Center for Disease Control and Prevention (CDC) U01DP006266 and U01DP003206; Association of Schools of Public Health/ CDC S043, S1734, S3486; and National Institutes of Health/National Institute of Arthritis and Musculoskeletal and Skin Diseases P60AR30701, P60AR049465, P60AR064166, and P30AR072580.

MOST: The MOST study was funded by the National Institutes of Health – National Institute on Aging grants AG19069 (Michael Nevitt, University of California, San Francisco) AG18820 (David Felson, Boston University) AG18947 (Cora Lewis, University of Alabama at Birmingham) and AG18832 (James Torner, University of Iowa).

OAI: The OAI is a public-private partnership comprised of five contracts (N01-AR-2-2258; N01-AR-2-2259; N01-AR-2-2260; N01-AR-2-2261; N01-AR-2-2262) funded by the National Institutes of Health, a branch of the Department of Health and Human Services, and conducted by the OAI Study Investigators. Private funding partners include Merck Research Laboratories; Novartis Pharmaceuticals Corporation, GlaxoSmithKline; and Pfizer, Inc.

Rotterdam Study: The Rotterdam Study is funded by Erasmus University Medical Center and Erasmus University, Rotterdam, The Netherlands Organisation for Health Research and

For peer review only

Development (ZonMw), the Research Institute for Diseases in the Elderly (RIDE), the Ministry of Education, Culture and Science, the Ministry for Health, Welfare and Sports, the European Commission (DG XII), and the Municipality of Rotterdam. The authors are grateful to the study participants, the staff from the Rotterdam Study and the participating general practitioners and pharmacists.

SOF: The Study of Osteoporotic Fractures (SOF) is supported by National Institutes of Health funding. The National Institute on Aging (NIA) provides support under the following grant numbers: R01 AG005407, R01 AR35582, R01 AR35583, R01 AR35584, R01 AG005394, R01 AG027574, and R01 AG027576

TASOAC: The TASOAC study was supported by the National Health and Medical Research Council of Australia, Tasmanian Community Fund, Masonic Centenary Medical Research Foundation, Royal Hobart Hospital Research Foundation and Arthritis Foundation of Australia.

Funding:

The World COACH consortium itself has been funded through research grants by the Dutch Arthritis Society (grant no. 18-2-203 and 21-1-205), the Dutch Research Council (NWO Veni grant scheme no. 09150161910071), and Erasmus MC, University Medical Center Rotterdam (Erasmus MC Fellowship).

MvB is funded by the Dutch Arthritis Society (research grants 18-2-203 and 21-1-205) and by an Erasmus MC Fellowship grant. NR, MvdB and FB are funded by the Dutch Arthritis Society (research grant 21-1-205). NA is funded by the Versus Arthritis Centre for Sport, Exercise & Osteoarthritis Research. SBZ is funded through independent research grants by

620 the European Research Council (ERC), the Dutch Arthritis Society, and The Netherlands Organisation for Health Research and Development (ZonMw). CB and JvM are funded by the Dutch Arthritis Society (LLP-34). TC is funded by research grants from the Engineering and Physical Sciences Research Council (EPSRC) UK, the Medical Research Council (MRC) UK, and the Wellcome Trust. KC and JH are funded by the National Health and Medical Research Council (NHMRC) Australia (GNT GNT1088683), AM is funded by an NHMRC Australia Early Career Fellowship (GNT1156674). DF is funded by a research grant from the National Institutes of Health (NIH) [AR072571]. CL is funded by a research grant from the MRC UK (MR/S00405X/1) as well as a Sir Henry Dale Fellowship jointly funded by the Wellcome Trust and the Royal Society (223267/Z/21/Z). This research was funded in whole, or in part, by the Wellcome Trust [Grant number 223267/Z/21/Z]. AN is funded by the Centers for Disease Control and Prevention (CDC) [U01DP006266 and U01DP003206; Association of Schools of Public Health/ CDC S043, S1734, S3486], and the NIH and National Institute of Arthritis and Musculoskeletal and Skin Diseases (NIAMS) [P60AR30701, P60AR049465, P60AR064166, and P30AR072580]. MN is funded by research grants from the NIH. JT is funded by the China Scholarship Council (CSC). HW is funded by research grants from Interreg, Kansen voor West, NWO, the Innovative Medicines Initiative (IMI), and the Dutch government. RA is funded by the Dutch Arthritis Society (research grants 18-2-203 and 21-1-205), the Dutch Research Council (NWO Veni grant scheme no. 09150161910071), and Erasmus MC, University Medical Center Rotterdam (Erasmus MC Fellowship).

Competing interests statement:

For peer review only

GJ reports personal fees from Novartis outside the submitted work. SBZ reports consulting fees from Pfizer Infirst Healthcare and personal fees for being a Deputy Editor for *Osteoarthritis and Cartilage* outside the submitted work. CL and TC report a patent for an image processing apparatus and method for fitting a deformable shape model to an image using random forest regression voting. CL reports licensing royalties for this patent from Optasia Medical outside the submitted work. AN is an associate editor for *Osteoarthritis and Cartilage* and is on the OARSI Board of Directors outside the submitted work. AM is on the Editorial Board for the *British Journal of Sports Medicine* and the *Journal of Science and Medicine in Sport* outside the submitted work. HW reports being a minority shareholder of Uplanner BV and Replasia BV outside the submitted work.

Contributors:

RA initiated the study. RA, MvB, NR, MvdB, FB, NA, SBZ, CB, FC, TC, DF, WPG, GJ, SK, NL, CL, JvM, AN, MN, JR, and HW worked on the conceptual design of the study. MvB and RA identified eligible cohorts and contacted cohort investigators for collaboration. MvB, RA, NR, MvdB, HA, KC, JH, SK, JL, JvM, AM, AN, MN, JT and HW collected the existing cohort data. MvB, NR, MvdB, FB, JT, and RA have worked on the database and on the harmonisation process. MvB, NR, MvdB, FB, VA, CB, TC, WPG, CL, JL, JvM, AN, MN, EO, JR, JT, and RA have worked on (preliminary) statistical analyses so far. MvB, NR, MvdB, FB, HA, VA, NA, SBZ, CB, FC, TC, KC, DF, WPG, JH, GJ, SK, NL, CL, L, JvM, AM, AN, MN, EO, JR, JT, HW, and RA critically reviewed and revised the manuscript and contributed to interpretation of the data. All authors read and approved the final version of the manuscript.

For peer review only

665 ## TABLES AND FIGURES

666

667 **Table 1: Information and inclusion criteria of the included cohorts.**

Cohort	Participants, inclusion criteria & recruitment	Age at enrolment	Races included	Follow-up time	Follow-up status
CHECK	Specific population: First visit to GP with hip/knee pain in the Netherlands<br>Recruited through advertisements, newspaper articles, flyers, GP referral, friends and family	45 – 65 years	W, B, A, O	10 years	Completed
Chingford	General population: asymptomatic women<br>Drawn from the register of a large general practice in Chingford, London, UK	45 – 67 years	NS	19 years	Completed
FORCe	Specific population: Sub-elite soccer and Australian football players with hip and/or groin pain and a physical exam indicative of femoroacetabular impingement<br>Recruited through advertisements, and from orthopaedic, sports medicine or physical therapy clinics	18 – 50 years	NS	5 years	Ongoing
JoCoOA	General population: Black and White men and women in a rural area<br>Drawn by probability sampling from the population of Johnston County, NC, USA	≥45 years	W, B	25+ years	Completed
MOST	Specific population: Individuals with existing knee OA or those at high risk for it<br>Identified through health insurance companies, voter registration tapes, commercial list brokers, and other sources<br>Recruited by mailings of letters and study brochures to eligible individuals	50 – 79 years	W, B, A, O, NS	7 years	Ongoing
OAI	Specific population: Individuals with existing knee OA or those at high risk for it<br>Recruited through focused mailings, advertisements in local newspapers, presentations at church, community or civic meetings, and a website about knee pain and osteoarthritis	45 – 79 years	W, B, A, O, NS	8 years	Completed
RS	General population: people living in the Ommoord district in Rotterdam, the Netherlands<br>Drawn from the municipal register, after which random clusters of potential participants got invited through a letter sent to their home	≥ 45 years	W, B, A, Mix	25 years	Ongoing
SOF	General population: community-dwelling women<br>Identified through health insurance companies, jury selection lists, voter registration lists, and drivers' licenses and identification cards lists<br>Recruited by mailings of letters and study brochures to eligible individuals	≥ 65 years	W, B	8 years	Completed
TASOAC	General population: community-dwelling men and women<br>Randomly selected from the electoral roll in Southern Tasmania	50 – 80 years	W, NS	10 years	Completed
**World COACH**	**Mix of individuals from the general population, those with possible early hip or knee OA, those with pre-existent knee OA or at high risk for it, and sub-elite**	**18 – 80 years**	**W, B, A, O, Mix**	**5 – 25 years**	**Ongoing**

For peer review only

	**football players with hip/groin pain and possible femoroacetabular impingement**				

CHECK = Cohort Hip and Cohort Knee; Chingford = Chingford 1000 women study; FORCe = Femoroacetabular Impingement and Hip Osteoarthritis Cohort Study; JoCoOA = Johnston County Osteoarthritis Project; MOST = Multicenter Osteoarthritis Study; OAI = Osteoarthritis Initiative; RS = The Rotterdam Study; SOF = Study of Osteoporotic Fractures; TASOAC = Tasmanian Older Adult Cohort; World COACH = Worldwide Collaboration on OsteoArthritis prediCtion for the Hip; Race abbreviations: W = white, B = black, A = Asian, O = other, NS = not specified, Mix = admixture

For peer review only

Table 2: Baseline characteristics of the included cohorts.

Cohort	N baseline participants	N participants with data available	N baseline pelvic / hip radiographs	Mean (SD) age, years	Mean (SD) height, cm	Mean (SD) weight, kg	Mean (SD) BMI, kg/m^2	% Female
CHECK	1,002	1,002	1,002	55.9 (5.2)	170 (8)	75.5 (13.8)	26.2 (4.0)	79%
Chingford	1,003	1,003	1,003	54.2 (6.0)	162 (6)	66.9 (11.8)	25.6 (4.3)	100%
FORCe	239	239	239	27.2 (5.9)	178 (9)	78.5 (12.8)	24.6 (3.1)	22%
JoCoOA	4,337	4,010	4,004	62.2 (10.0)	166 (10)	81.4 (18.3)	29.5 (6.2)	62%
MOST	3,026	3,026	3,008	62.5 (8.1)	169 (12)	87.9 (18.9)	30.7 (6.0)	60%
OAI	4,796	4,796	4,771	61.2 (9.2)	165 (24)	81.2 (16.4)	28.6 (4.8)	58%
RS	14,926	14,926	11,147	66.0 (10.5)	168 (10)	75.9 (13.9)	26.9 (4.1)	59%
SOF	10,366	9,704	8,291	71.6 (5.2)	159 (6)	67.0 (12.0)	26.4 (4.5)	100%
TASOAC	1,099	1,099	1,099	63.0 (7.5)	167 (9)	77.9 (15.0)	27.9 (4.8)	51%
**World COACH**	**40,794**	**39,805**	**34,257**	**65.4 (8.8)**	**166 (12)**	**75.7 (14.7)**	**27.5 (4.7)**	**70%**

CHECK = Cohort Hip and Cohort Knee; Chingford = Chingford 1000 women study; FORCe = Femoroacetabular Impingement and Hip Osteoarthritis Cohort Study; JoCoOA = Johnston County Osteoarthritis Project; MOST = Multicenter Osteoarthritis Study; OAI = Osteoarthritis Initiative; RS = The Rotterdam Study; SOF = Study of Osteoporotic Fractures; TASOAC = Tasmanian Older Adult Cohort; World COACH = Worldwide Collaboration on OsteoArthritis prediCtion for the Hip; SD = Standard deviation; BMI = body mass index

For peer review only

Table 3: Indication of radiographic hip osteoarthritis scores at baseline

Cohort	N total hips	Available RHOA scores	0	1	2	3	4	THR	N Missing
CHECK	2,004	KL, (OARSI)	1,225	458	204	13	0	0	104
CHINGFORD	2,006	KL, (OARSI)	1,031	78	152	5	1	1	738
FORCe	465	KL	446	19	0	0	0	0	0
JOCO	8,020	KL, (OARSI)	1,837	4,286	1,493	91	43	0	270
MOST	6,052	KL, (OARSI)	2,194	1,004	354	75	7	0	2,418
OAI	9,592	OA score (based on modified Croft) , OARSI	7,121	1,064	543	*NA*	*NA*	*NA*	864
RS1	16,240	KL, (OARSI)	6,406	4,110	426	63	18	216	5,001
RS2	6,024	KL, (OARSI)	3,584	669	105	15	5	62	1,584
RS3	7,878	KL, (OARSI)	5,282	532	58	4	2	42	1,958
SOF	19,414	Modified Croft, (OARSI)	5,725	5,558	262	117	10	0	7,742
TASOAC	1,962	OA score (based on OARSI), (OARSI)	1,214	*NA*	748	*NA*	*NA*	*NA*	0
**World COACH**	79,657	Mix	36,065	17,778	4,345	383	86	321	20,679

Scores 0-4 are either KL, modified Croft, or a custom summary OA score. OARSI scores are not shown in this table.

CHECK = Cohort Hip and Cohort Knee; Chingford = Chingford 1000 women study; FORCe = Femoroacetabular Impingement and Hip Osteoarthritis Cohort Study; JoCoOA = Johnston County Osteoarthritis Project; MOST = Multicenter Osteoarthritis Study; OAI = Osteoarthritis Initiative; RS = The Rotterdam Study; SOF = Study of Osteoporotic Fractures; TASOAC = Tasmanian Older Adult Cohort; RHOA = radiographic hip osteoarthritis; World COACH = Worldwide Collaboration on OsteoArthritis prediCtion for the Hip; THR = total hip replacement, KL = Kellgren & Lawrence, OARSI = Osteoarthritis Research Society International individual features scores; NA = not applicable.

For peer review only

Figure 1: PRISMA flow diagram detailing the literature search, screening, and inclusion process for the World COACH consortium.

PRISMA, Preferred Reporting Items for Systematic Reviews and Meta-Analyses; World COACH, Worldwide Collaboration on OsteoArthritis prediCtion for the Hip.

Figure 2: A simplified schema of the relational database used in the World COACH consortium.

World COACH, Worldwide Collaboration on OsteoArthritis prediCtion for the Hip.

For peer review only

References

1. Hunter DJ, Bierma-Zeinstra S. Osteoarthritis. *Lancet* 2019;393(10182):1745-1759. PMID: 31034380 doi: 10.1016/s0140-6736(19)30417-9.
2. GBD 2015 Disease and Injury Incidence and Prevalence Collaborators. Global, regional, and national incidence, prevalence, and years lived with disability for 310 diseases and injuries, 1990-2015: a systematic analysis for the Global Burden of Disease Study 2015. *Lancet* 2016;388(10053):1545-1602. PMID: 27733282 doi: 10.1016/s0140-6736(16)31678-6.
3. Gupta S, Hawker GA, Laporte A, et al. The economic burden of disabling hip and knee osteoarthritis (OA) from the perspective of individuals living with this condition. *Rheumatology (Oxford, England)* 2005;44(12):1531-1537. PMID: 16091394 doi: 10.1093/rheumatology/kei049.
4. Hunter DJ, Schofield D, Callander E. The individual and socioeconomic impact of osteoarthritis. *Nature Reviews Rheumatology* 2014;10(7):437-441. PMID: 24662640 doi: 10.1038/nrrheum.2014.44.
5. Clynes MA, Jameson KA, Edwards MH, et al. Impact of osteoarthritis on activities of daily living: does joint site matter? *Aging Clin Exp Res* 2019;31(8):1049-1056. PMID: 30903599 doi: 10.1007/s40520-019-01163-0.
6. Katz JN, Arant KR, Loeser RF. Diagnosis and Treatment of Hip and Knee Osteoarthritis: A Review. *JAMA* 2021;325(6):568-578. PMID: 33560326.
7. Bannuru RR, Osani MC, Vaysbrot EE, et al. OARSI guidelines for the non-surgical management of knee, hip, and polyarticular osteoarthritis. *Osteoarthritis Cartilage* 2019;27(11):1578-1589. PMID: 31278997 doi: 10.1016/j.joca.2019.06.011.
8. Appleyard T, Thomas MJ, Antcliff D, et al. Prediction Models to Estimate the Future Risk of Osteoarthritis in the General Population: A Systematic Review. *Arthritis Care Res (Hoboken)* 2023;75(7):1481-1493. PMID: 36205228.
9. Karlson EW, Mandl LA, Aweh GN, et al. Total hip replacement due to osteoarthritis: the importance of age, obesity, and other modifiable risk factors: Elsevier 2003.
10. Rodriguez-Fontenla C, Calaza M, Evangelou E, et al. Assessment of osteoarthritis candidate genes in a meta-analysis of nine genome-wide association studies. *Arthritis Rheumatol* 2014;66(4):940-949. PMID: 24757145 doi: 10.1002/art.38300.
11. Jordan JM, Helmick CG, Renner JB, et al. Prevalence of hip symptoms and radiographic and symptomatic hip osteoarthritis in African Americans and Caucasians: the Johnston County Osteoarthritis Project. *J Rheumatol* 2009;36(4):809-815. PMID: 19286855.
12. Casartelli NC, Maffiuletti NA, Valenzuela PL, et al. Is hip morphology a risk factor for developing hip osteoarthritis? A systematic review with meta-analysis. *Osteoarthritis Cartilage* 2021;29(9):1252-1264. PMID: 34171473 doi: S1063-4584(21)00813-X [pii]

10.1016/j.joca.2021.06.007.

13. van Buuren MMA, Arden NK, Bierma-Zeinstra SMA, et al. Statistical shape modeling of the hip and the association with hip osteoarthritis: a systematic review. *Osteoarthritis Cartilage* 2021;29(5):607-618. PMID: 33338641 doi: 10.1016/j.joca.2020.12.003.

14. Xue Y, Zhang R, Deng Y, et al. A preliminary examination of the diagnostic value of deep learning in hip osteoarthritis. *PLoS One* 2017;12(6):e0178992.

15. von Schacky CE, Sohn JH, Liu F, et al. Development and Validation of a Multitask Deep Learning Model for Severity Grading of Hip Osteoarthritis Features on Radiographs. *Radiology* 2020;295(1):136-145. PMID: 32013791 doi: 10.1148/radiol.2020190925.

16. Doiron D, Burton P, Marcon Y, et al. Data harmonization and federated analysis of population-based studies: the BioSHaRE project. *Emerging Themes in Epidemiology* 2013;10(1):7622-7610-7612. PMID: 24257327 doi: 10.1186/1742-7622-10-12.

17. Crossley KM, Pandy MG, Majumdar S, et al. Femoroacetabular impingement and hip OsteoaRthritis Cohort (FORCe): protocol for a prospective study. *Journal of Physiotherapy* 2018;64(1):55. PMID: 29289588 doi: S1836-9553(17)30136-4 [pii]
10.1016/j.jphys.2017.10.004.

18. Cummings SR, Black DM, Nevitt MC, et al. Appendicular bone density and age predict hip fracture in women. The Study of Osteoporotic Fractures Research Group. *JAMA* 1990;263(5):665-668. PMID: 2404146.

19. Hart DJ, Spector TD. Cigarette smoking and risk of osteoarthritis in women in the general population: the Chingford study. *Ann Rheum Dis* 1993;52(2):93-96. PMID: 8447703 doi: 10.1136/ard.52.2.93.

20. Ikram MA, Brusselle GGO, Murad SD, et al. The Rotterdam Study: 2018 update on objectives, design and main results. *European Journal of Epidemiology* 2017;32(9):807-850. PMID: 29064009 doi: 10.1007/s10654-017-0321-4.

21. Laslett LL, Quinn SJ, Winzenberg TM, et al. A prospective study of the impact of musculoskeletal pain and radiographic osteoarthritis on health related quality of life in community dwelling older people. *BMC Musculoskelet Disord* 2012;13(Journal Article):2474-2413-2168. PMID: 22954354 doi: 10.1186/1471-2474-13-168.

22. Lester G. Clinical research in OA--the NIH Osteoarthritis Initiative. *J Musculoskelet Neuronal Interact* 2008;8(4):313-314. PMID: 19147953.

23. Nelson AE, Hu D, Arbeeva L, et al. Point prevalence of Hip Symptoms, Radiographic, And Symptomatic OA at Five Time Points: The Johnston County Osteoarthritis Project, 1991-2018. *Osteoarthritis and Cartilage Open* 2022;4(2) PMID: 36118130 doi: 100251 [pii]
10.1016/j.ocarto.2022.100251.

24. Segal NA, Nevitt MC, Gross KD, et al. The Multicenter Osteoarthritis Study: opportunities for rehabilitation research. *PM & R : the journal of injury, function, and rehabilitation* 2013;5(8):647-654. PMID: 23953013 doi: 10.1016/j.pmrj.2013.04.014.

25. Wesseling J, Boers M, Viergever MA, et al. Cohort Profile: Cohort Hip and Cohort Knee (CHECK) study. *International Journal of Epidemiology* 2016;45(1):36-44. PMID: 25172137 doi: 10.1093/ije/dyu177.

For peer review only

26. Iidaka T, Horii C, Muraki S, et al. Trends in prevalence of hip osteoarthritis over a 10-year period in Japan: The ROAD study 2005-2015. *Osteoarthr Cartil Open* 2022;4(3):100285. PMID: 36474937.
27. Nicholls AS, Kiran A, Pollard TCB, et al. The association between hip morphology parameters and nineteen-year risk of end-stage osteoarthritis of the hip: A nested case-control study. *Arthritis Rheum* 2011;63(11):3392-3400. doi: 10.1002/art.30523.
28. Jordan JM, Linder GF, Renner JB, et al. The impact of arthritis in rural populations. *Arthritis Care and Research* 1995;8(4):242-250. PMID: 8605262 doi: 10.1002/art.1790080407.
29. Nevitt MC, Felson DT, Lester G. The Osteoarthritis Initiative. Protocol for the cohort study. ;V 1.1 6.21.06. *National Institute of Arthritis, Musculoskeletal and Skin Diseases*.
30. Ikram MA, Brusselle G, Ghanbari M, et al. Objectives, design and main findings until 2020 from the Rotterdam Study. *Eur J Epidemiol* 2020;35(5):483-517. PMID: 32367290.
31. Ahedi H, Aitken D, Blizzard L, et al. Quantification of hip effusion-synovitis and its cross-sectional and longitudinal associations with hip pain, MRI findings and early radiographic hip OA. *BMC Musculoskelet Disord* 2020;21(1):020-03532-03537. PMID: 32778082 doi: 10.1186/s12891-020-03532-7.
32. Fortier I, Raina P, Van den Heuvel ER, et al. Maelstrom Research guidelines for rigorous retrospective data harmonization. *International Journal of Epidemiology* 2017;46(1):103-105. PMID: 27272186 doi: 10.1093/ije/dyw075.
33. Stang PE, Ryan Pb Fau - Racoosin JA, Racoosin Ja Fau - Overhage JM, et al. Advancing the science for active surveillance: rationale and design for the Observational Medical Outcomes Partnership. *Annals of Internal Medicine* 2010;153(1539-3704 (Electronic)):600-606. PMID: 21041580 doi: 153/9/600 [pii]
10.7326/0003-4819-153-9-201011020-00010.
34. Overhage JM, Ryan PB, Reich CG, et al. Validation of a common data model for active safety surveillance research. *Journal of the American Medical Informatics Association* 2012;19(1):54-60. PMID: 22037893 doi: amiajnl-2011-000376 [pii]
10.1136/amiajnl-2011-000376.
35. Altman RD, Bloch DA, Dougados M, et al. Measurement of structural progression in osteoarthritis of the hip: the Barcelona consensus group. *Osteoarthritis Cartilage* 2004;12(7):515-524. PMID: 15219566 doi: 10.1016/j.joca.2004.04.004.
36. Gold GE, Cicuttini F, Crema MD, et al. OARSI Clinical Trials Recommendations: Hip imaging in clinical trials in osteoarthritis. *Osteoarthritis Cartilage* 2015;23(5):716-731. PMID: 25952344 doi: 10.1016/j.joca.2015.03.004.
37. Kellgren JH, Lawrence JS. Radiological assessment of osteo-arthrosis. *Ann Rheum Dis* 1957;16(4):494-502. PMID: 13498604 doi: 10.1136/ard.16.4.494.
38. Macri EM, Runhaar J, Damen J, et al. Kellgren/Lawrence Grading in Cohort Studies: Methodological Update and Implications Illustrated Using Data From a Dutch Hip and Knee Cohort. *Arthritis Care and Research* 2022;74(7):1179-1187. PMID: 33450140 doi: 10.1002/acr.24563.
39. Nevitt MC, Lane NE, Scott JC, et al. Radiographic osteoarthritis of the hip and bone mineral density. *Arthritis Rheum* 1995;38(7):907-916. PMID: 7612040 doi: 10.1002/art.1780380706.

For peer review only

40. Croft P, Cooper C, Wickham C, et al. Defining osteoarthritis of the hip for epidemiologic studies. *American Journal of Epidemiology* 1990;132(3):514-522. PMID: 2389755 doi: 10.1093/oxfordjournals.aje.a115687.

41. Lane NE, Nevitt MC, Hochberg MC, et al. Progression of radiographic hip osteoarthritis over eight years in a community sample of elderly white women. *Arthritis Rheum* 2004;50(5):1477-1486. PMID: 15146417 doi: 10.1002/art.20213.

42. Altman RD, Gold GE. Atlas of individual radiographic features in osteoarthritis, revised. *Osteoarthritis Cartilage* 2007;15:A1-A56. PMID: 17320422 doi: S1063-4584(06)00328-1 [pii]
10.1016/j.joca.2006.11.009.

43. Kolasinski SL, Neogi T, Hochberg MC, et al. 2019 American College of Rheumatology/Arthritis Foundation Guideline for the Management of Osteoarthritis of the Hand, Hip, and Knee. *Arthritis Care Res (Hoboken)* 2020;72(2):149-162. PMID: 31908149.

44. NICE. Osteoarthritis in over 16s: diagnosis and management. *National Institute for Health and Care Excellence: Guidelines* 2022 PMID: 36745715.

45. Sakellariou G, Conaghan PG, Zhang W, et al. EULAR recommendations for the use of imaging in the clinical management of peripheral joint osteoarthritis. *Ann Rheum Dis* 2017;76(9):1484-1494. PMID: 28389554.

46. Wilkinson MD, Dumontier M, Aalbersberg IJ, et al. The FAIR Guiding Principles for scientific data management and stewardship. *Sci Data* 2016;3:160018. PMID: 26978244.

For peer review only

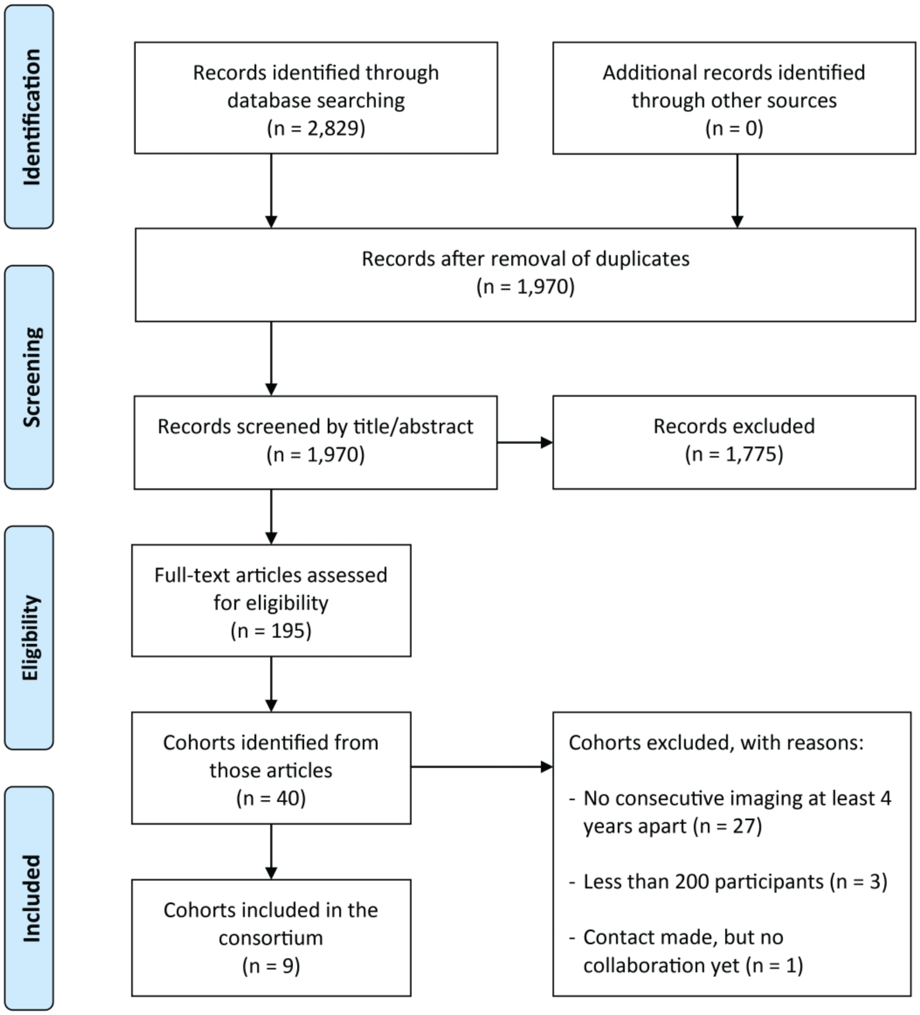

Figure 1: PRISMA flow diagram detailing the literature search, screening, and inclusion process for the World COACH consortium.

PRISMA, Preferred Reporting Items for Systematic Reviews and Meta-Analyses; World COACH, Worldwide Collaboration on OsteoArthritis prediCtion for the Hip.

434x474mm (236 x 236 DPI)

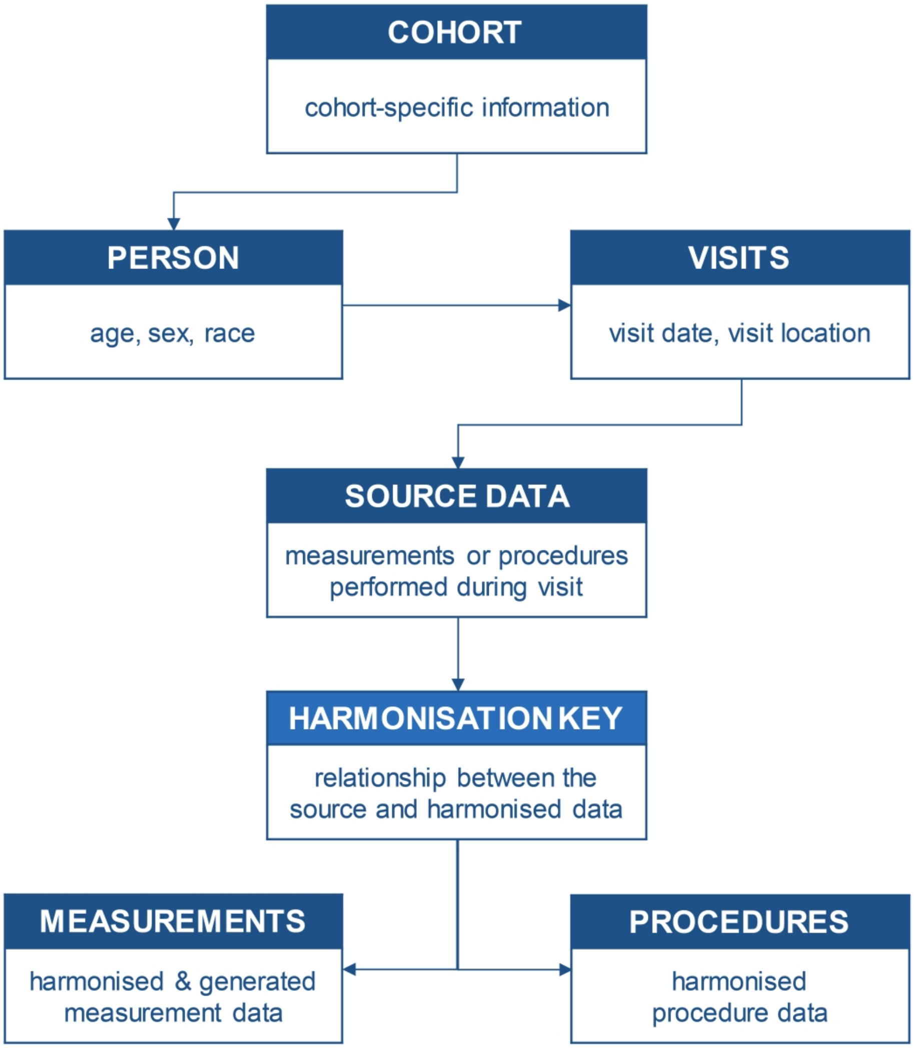

Figure 2: A simplified schema of the relational database used in the World COACH consortium.

World COACH, Worldwide Collaboration on OsteoArthritis prediCtion for the Hip.

422x480mm (236 x 236 DPI)

Supplement to:

Cohort profile: Worldwide Collaboration on OsteoArthritis prediCtion for the Hip (World COACH); an international consortium of prospective cohort studies with individual participant data on hip osteoarthritis.

Supplemental Table S1: Indication of available radiographic hip osteoarthritis scores in the included studies at different time points

	N hips with radiographic hip osteoarthritis score currently available for each follow-up time point										
**Cohort**	**Baseline**	**2 years**	**4 years**	**5 years**	**6 years**	**7 years**	**8 years**	**10 years**	**12 years**	**15 years**	**20+ years**
CHECK	1,900	1,848		1,746			1,689	1,650			
Chingford	1,268					1,265					x
FORCe	465			x							
JoCoOA	7,750			4,394				2,607		1,355	491
MOST	3,634			3,914							
OAI	8,728		6,712				x				
RS-I	11,239		x				6,929		4,911		2,865
RS-II	4,440		3,314						3,028	x	
RS-III	5,920				4,320						
SOF	11,672						11,672				
TASOAC	1,962							x			
**World COACH**	**58,978**	**1,848**	**10,026**	**10,054**	**4,320**	**1,265**	**20,290**	**4,257**	**7,939**	**1,355**	**3,356**

CHECK = Cohort Hip and Cohort Knee; Chingford = Chingford 1000 women study; FORCe = Femoroacetabular Impingement and Hip Osteoarthritis Cohort Study; JoCoOA = Johnston County Osteoarthritis Project; MOST = Multicenter Osteoarthritis Study; OAI = Osteoarthritis Initiative; RS = The Rotterdam Study; SOF = Study of Osteoporotic Fractures; TASOAC = Tasmanian Older Adult Cohort; World COACH = Worldwide Collaboration on OsteoArthritis prediCtion for the Hip; x = Radiographs available, but not yet scored or score not available yet

For peer review only

Supplemental Table S2: Indication of radiographic hip osteoarthritis development in the included studies

Cohort	Total hips, N	Hips with baseline score and at least 1 follow-up, N (%)	Hips without RHOA at baseline, N (%)	Hips with RHOA at baseline, N (%)	Hips without incident RHOA at follow-up, N (%)	Hips with incident RHOA at follow-up, N (%)
CHECK	2,004	1,827 (91%)	1,627 (89%)	200 (11%)	960 (59%)	667 (41%)
Chingford	2,006	1,265 (63%)	1,108 (88%)	157 (12%)	994 (90%)	114 (10%)
FORCe	465	*NA*	465 (100%)	0 (0%)	*NA*	*NA*
JoCoOA	8,020	4,385 (55%)	3,538 (81%)	847 (19%)	2,792 (79%)	746 (21%)
MOST	6,052	3,885 (64%)	3,486 (90%)	399 (10%)	3,334 (96%)	152 (4%)
OAI	9,592	6,669 (70%)	6,335 (95%)	334 (5%)	6,240 (99%)	95 (1%)
RS-I	16,240	7,192 (44%)	6,783 (94%)	409 (6%)	6,189 (91%)	594 (9%)
RS-II	6,024	3,316 (55%)	3,193 (96%)	123 (4%)	2,943 (92%)	250 (8%)
RS-III	7,878	3,638 (46%)	3,587 (99%)	51 (1%)	3,435 (96%)	152 (4%)
SOF	19,414	11,672 (60%)	11,283 (97%)	389 (3%)	10,846 (96%)	437 (4%)
TASOAC	1,962	*NA*	1,214 (62%)	748 (38%)	*NA*	*NA*
**World COACH**	**79,657**	**43,849 (57%)***	**42,619 (92%)**	**3,657 (8%)**	**37,733 (92%)***	**3,207 (8%)***

*FORCe and TASOAC are not included in this World COACH total for this column, so subtotals may not add up
CHECK = Cohort Hip and Cohort Knee; Chingford = Chingford 1000 women study; FORCe = Femoroacetabular Impingement and Hip Osteoarthritis Cohort Study; JoCoOA = Johnston County Osteoarthritis Project; MOST = Multicenter Osteoarthritis Study; OAI = Osteoarthritis Initiative; RS = The Rotterdam Study; SOF = Study of Osteoporotic Fractures; TASOAC = Tasmanian Older Adult Cohort; World COACH = Worldwide Collaboration on OsteoArthritis prediCtion for the Hip; RHOA = radiographic hip osteoarthritis; NA = Not applicable (no follow-up RHOA scores yet).

For peer review only
